# Pan-cancer analysis of pre-diagnostic blood metabolite concentrations in the European Prospective Investigation into Cancer and Nutrition

**DOI:** 10.1186/s12916-022-02553-4

**Published:** 2022-10-19

**Authors:** Marie Breeur, Pietro Ferrari, Laure Dossus, Mazda Jenab, Mattias Johansson, Sabina Rinaldi, Ruth C. Travis, Mathilde His, Tim J. Key, Julie A. Schmidt, Kim Overvad, Anne Tjønneland, Cecilie Kyrø, Joseph A. Rothwell, Nasser Laouali, Gianluca Severi, Rudolf Kaaks, Verena Katzke, Matthias B. Schulze, Fabian Eichelmann, Domenico Palli, Sara Grioni, Salvatore Panico, Rosario Tumino, Carlotta Sacerdote, Bas Bueno-de-Mesquita, Karina Standahl Olsen, Torkjel Manning Sandanger, Therese Haugdahl Nøst, J. Ramón Quirós, Catalina Bonet, Miguel Rodríguez Barranco, María-Dolores Chirlaque, Eva Ardanaz, Malte Sandsveden, Jonas Manjer, Linda Vidman, Matilda Rentoft, David Muller, Kostas Tsilidis, Alicia K. Heath, Hector Keun, Jerzy Adamski, Pekka Keski-Rahkonen, Augustin Scalbert, Marc J. Gunter, Vivian Viallon

**Affiliations:** 1 Nutrition and Metabolism Branch, International Agency for Research on Cancer, NME Branch, 69372 CEDEX 08 Lyon, France; 2 Genetics Branch, International Agency for Research on Cancer, 69372 CEDEX 08 Lyon, France; 3 Cancer Epidemiology Unit, Nuffield Department of Population Health, University of Oxford, Oxford, OX3 7LF UK; 4 Department of Clinical Epidemiology, Department of Clinical Medicine, Aarhus University Hospital and Aarhus University, DK-8200 Aarhus N, Denmark; 5 Department of Public Health, Aarhus University, DK-8000 Aarhus C, Denmark; 6 Danish Cancer Society Research Center Diet, Genes and Environment Nutrition and Biomarkers, DK-2100 Copenhagen, Denmark; 7 Université Paris-Saclay, UVSQ, Inserm, CESP U1018, “Exposome and Heredity” team, Gustave Roussy, 94800 Villejuif, France; 8 Division of Cancer Epidemiology, German Cancer Research Center (DKFZ), 69120 Heidelberg, Germany; 9 Department of Molecular Epidemiology, German Institute of Human Nutrition, 14558 Nuthetal, Germany; 10 German Center for Diabetes Research (DZD), 85764 Neuherberg, Germany; 11 Institute of Cancer Research, Prevention and Clinical Network (ISPRO), 50139 Florence, Italy; 12 Epidemiology and Prevention Unit, Fondazione IRCCS Istituto Nazionale dei Tumori di Milano, 20133 Milan, Italy; 13 Dipartimento di Medicina Clinica e Chirurgia, Federico II University, 80131 Naples, Italy; 14 Hyblean Association for Epidemiological Research, AIRE-ONLUS, 97100 Ragusa, Italy; 15 Unit of Cancer Epidemiology Città della Salute e della Scienza University-Hospital, 10126 Turin, Italy; 16 Centre for Nutrition, Prevention and Health Services, National Institute for Public Health and the Environment (RIVM), PO Box 1, 3720 BA Bilthoven, The Netherlands; 17 Department of Community Medicine, UiT The Arctic University of Norway, N-9037 Tromsø, Norway; 18 Public Health Directorate, 33006 Oviedo, Asturias Spain; 19 Unit of Nutrition and Cancer, Cancer Epidemiology Research Program, Catalan Institute of Oncology (ICO), Bellvitge Biomedical Research Institute (IDIBELL), L’Hospitalet de Llobregat, 08908 Barcelona, Spain; 20 Escuela Andaluza de Salud Pública (EASP), 18011 Granada, Spain; 21 Instituto de Investigación Biosanitaria ibs. GRANADA, 18012 Granada, Spain; 22 Centro de Investigación Biomédica en Red de Epidemiología y Salud Pública (CIBERESP), 28029 Madrid, Spain; 23 Department of Epidemiology, Regional Health Council, IMIB-Arrixaca, Murcia University, 30003 Murcia, Spain; 24 Navarra Public Health Institute, 31003 Pamplona, Spain; 25 IdiSNA, Navarra Institute for Health Research, 31008 Pamplona, Spain; 26 Department of Clinical Sciences Malmö Lund University, SE-214 28 Malmö, Sweden; 27 Departement of Surgery, Skåne University Hospital Malmö, Lund University, SE-214 28 Malmö, Sweden; 28 Department of Radiation Sciences, Oncology Umeå University, SE-901 87 Umeå, Sweden; 29 Department of Epidemiology and Biostatistics, School of Public Health, Imperial College London, London, W2 1PG UK; 30 Department of Surgery and Cancer, Cancer Metabolism and Systems Toxicology Group, Division of Cancer, Imperial College London, London, SW7 2AZ UK; 31 Institute of Experimental Genetics, Helmholtz Zentrum München, German Research Center for Environmental Health, 85764 Neuherberg, Germany; 32 Department of Biochemistry, Yong Loo Lin School of Medicine, National University of Singapore, Singapore, 117597 Singapore; 33 Institute of Biochemistry, Faculty of Medicine, University of Ljubljana, 1000 Ljubljana, Slovenia

**Keywords:** Metabolomics, Cancer, Breast, Colorectal, Endometrial, Kidney, Liver, Prostate, Lasso, EPIC

## Abstract

**Background:**

Epidemiological studies of associations between metabolites and cancer risk have typically focused on specific cancer types separately. Here, we designed a multivariate pan-cancer analysis to identify metabolites potentially associated with multiple cancer types, while also allowing the investigation of cancer type-specific associations.

**Methods:**

We analysed targeted metabolomics data available for 5828 matched case-control pairs from cancer-specific case-control studies on breast, colorectal, endometrial, gallbladder, kidney, localized and advanced prostate cancer, and hepatocellular carcinoma nested within the European Prospective Investigation into Cancer and Nutrition (EPIC) cohort. From pre-diagnostic blood levels of an initial set of 117 metabolites, 33 cluster representatives of strongly correlated metabolites and 17 single metabolites were derived by hierarchical clustering. The mutually adjusted associations of the resulting 50 metabolites with cancer risk were examined in penalized conditional logistic regression models adjusted for body mass index, using the data-shared lasso penalty.

**Results:**

Out of the 50 studied metabolites, (i) six were inversely associated with the risk of most cancer types: glutamine, butyrylcarnitine, lysophosphatidylcholine a C18:2, and three clusters of phosphatidylcholines (PCs); (ii) three were positively associated with most cancer types: proline, decanoylcarnitine, and one cluster of PCs; and (iii) 10 were specifically associated with particular cancer types, including histidine that was inversely associated with colorectal cancer risk and one cluster of sphingomyelins that was inversely associated with risk of hepatocellular carcinoma and positively with endometrial cancer risk.

**Conclusions:**

These results could provide novel insights for the identification of pathways for cancer development, in particular those shared across different cancer types.

**Supplementary Information:**

The online version contains supplementary material available at 10.1186/s12916-022-02553-4.

## Background

Metabolomics allows the simultaneous measurement of a large variety of compounds present in biological samples, such as human blood [1, 2]. Circulating metabolite levels can reflect both endogenous and exogenous processes, providing a snapshot of biological activity [3, 4]. As a result, metabolomics may facilitate the identification of biological mechanisms involved in the development of chronic diseases. For example, prior metabolomics studies have identified metabolites associated with the risk of various chronic conditions, including type 2 diabetes (T2D) [5–7], cardiovascular diseases (CVD) [8–10], and different site-specific cancers, including cancers of the breast [11], prostate [12, 13], endometrium [14], kidney [15], colorectum [16–18], hepatocellular carcinoma (HCC) [19], and others [20, 21].

Several shared biological mechanisms are known to underlie multiple chronic diseases. Obesity, physical inactivity, and adherence to a Western-type diet, as well as chronic inflammation and insulin resistance, are recognized risk factors for cardio-metabolic diseases, including T2D, CVD, and several site-specific cancers [22–24]. Metabolomics may help uncover novel etiological mechanisms that are common to several chronic diseases as well as those that are disease-specific. One recent study identified metabolites associated with the risk of multimorbidity, defined as the simultaneous presence of multiple chronic conditions within one individual [25]. Focusing on a pre-defined panel of metabolites, a targeted metabolomics study of breast, prostate, and colorectal cancers in a German population found that circulating levels of the phosphatidylcholine PC ae C30:0 and several lysophosphatidylcholines, including lysoPC a C18:0, were predictive of the development of any of these three cancers [26], suggesting that some etiological mechanisms could be shared across multiple cancer types.

In this work, we extended this concept by leveraging targeted metabolomics data available within nested case-control studies on eight cancer types (breast, colorectal, endometrial, gallbladder and biliary tract, kidney, localized prostate and advanced prostate cancers, and HCC) previously acquired in the European Prospective Investigation into Cancer and Nutrition (EPIC) [11, 12, 14, 15, 19]. The data-shared lasso [27–29], a penalized multivariate approach specifically designed for the investigation of a set of shared risk factors across different disease outcomes, was used to carry out a multivariate pan-cancer analysis to identify mutually adjusted metabolites associated with cancer risk and to identify those metabolites with consistent or heterogeneous patterns of associations across the eight cancer types.

## Methods

### Study population

EPIC is an ongoing multicentric prospective study with over 500,000 men and women recruited between 1992 and 2000 from 23 centres in 10 European countries [30], originally designed to study the relationship between diet and cancer risk. Incident cancer cases were identified through a combination of methods, including health insurance records, cancer and pathology registries, and active follow-up through study participants and their next-of-kin. At recruitment, information on diet and lifestyle was collected via self-administered questionnaires. Blood samples were collected from around 386,000 participants according to a standardized protocol. In France, Germany, Greece, Italy, the Netherlands, Norway, Spain, and the UK, serum (except in Norway), plasma, erythrocytes, and buffy coat aliquots were stored in liquid nitrogen (− 196 °C) in a centralized biobank at the International Agency for Research on Cancer (IARC). In Denmark, blood fractions were stored locally in the vapour phase of liquid nitrogen containers (− 150 °C), and in Sweden, they were stored locally at − 80 °C in standard freezers. Fasting was not required.

Our analyses used a set of metabolomics measurements from 15,948 EPIC participants from seven cancer-specific matched case-control studies nested within EPIC (Table 1). In each study, each case was matched to one control selected among cancer-free participants (other than non-melanoma skin cancer) by risk set sampling, using matching factors that included study centre, sex, age at blood collection, time of the day of blood collection, fasting status, and use of exogenous hormones for women.

**Table 1:** Description of the original seven cancer-specific matched case-control studies nested within EPIC

Cancer site	Number of samples	Matrix	Laboratory	Kit used
Breast	3172	Citrate plasma^a^	IARC	p180
Colorectal (study 1)	946	Citrate plasma	IARC	p180
Colorectal (study 2)	2295	Serum	HZM^b^	p150
Endometrial	1706	Citrate plasma	ICL^c^	p180
Liver	662	Serum	IARC	p180
Kidney	1213	Citrate plasma	IARC	p180
Prostate	6020	Citrate plasma	IARC	p180

^a^Except Swedish participants (*n*=101; EDTA plasma)

^b^Helmhotz Zentrum München

^c^Imperial College London

All participants provided written informed consent to participate in the EPIC study. The cancer-specific case-control studies were all approved by the ethics committee of IARC and participating EPIC centres.

### Laboratory analysis

As summarized in Table 1, pre-diagnostic blood samples were assayed at the Helmholtz Zentrum (München, Germany) for the second colorectal cancer study, at Imperial College London (UK) for the endometrial cancer study, and at IARC for all other studies. Data for a total of 171 metabolites were acquired by tandem mass spectrometry using either the AbsoluteIDQ p150 (for the second colorectal cancer study) or the AbsoluteIDQ p180 commercial kit (Biocrates Life Science AG, Innsbruck Austria). Two successive assays were used, liquid chromatography-tandem mass spectrometry (LC-MS/MS) for amino acids and biogenic amines, and flow injection analysis-tandem mass spectrometry (FIA-MS/MS) for the other metabolites. Samples were either serum or citrate plasma, and samples within each study were all from the same type of blood matrix, except for the breast cancer study (Table 1). Samples of each case-control pair were assayed on the same batch (and in the same laboratory).

### Selection of the metabolites and data pre-processing

Data were pre-processed following an established procedure [31]. Briefly, metabolites with more than 25% missing values in any study were excluded. Samples with more than 25% missing values overall were excluded, as were those detected as outliers by a principal component analysis (PCA)-based approach applied within each study separately. Then, for all metabolites measured by FIA with a semi-quantitative method (acylcarnitines, glycerophospholipids, sphingolipids, hexoses), measurements below the batch-specific limit of detection (LOD) were imputed to half the LOD. When the batch-specific LOD was unknown, LOD was first set to study-specific medians of known batch-specific LODs. For the metabolites measured with a fully quantitative approach (amino acids and biogenic amines), measurements below the lower limit of quantification (LLOQ) or above the upper limit of quantification (ULOQ) were imputed to half the LLOQ or to the ULOQ, respectively. For all metabolites, other missing values were imputed to the batch-specific median of the non-missing measurements. The resulting measurements were then log-transformed to improve symmetry.

### Cancer types and exclusion criteria

We focused on eight cancer types, namely breast, colorectal, endometrial, kidney, gallbladder and biliary tract cancers, HCC, and advanced and localized prostate. As detailed in the Supplementary Material (Additional file 1: Section 1 [12, 19]), matched case-control pairs for HCC and gallbladder and biliary tract cancer were extracted from the liver cancer study, while matched case-control pairs for advanced and localized prostate cancer were extracted from the prostate cancer study. Since hormones could affect metabolite levels and their association with cancer risk [11], women using exogenous hormones (either hormone replacement therapy or oral contraceptive) at baseline were excluded.

### Statistical analyses

All analyses were performed using R software. Characteristics of cases and controls for the eight studied cancer types were described using the mean and standard deviation or frequency. Pearson correlations between the metabolites were computed in controls only to reduce collider bias.

#### Clustering of metabolites

The most strongly correlated metabolites were grouped together by applying the hierarchical clustering approach implemented in the ClustOfVar R package [32] to the control samples. For each cluster, the method defined its representative as the first principal component in the PCA of the metabolites grouped into that cluster. In our figures and tables, cluster representatives were labelled as “xxx_clus”, with “xxx” representing one particular metabolite that composed that cluster. We retained the model with the lowest number of clusters such that representatives explained at least 80% of the total variation in each cluster. Cluster representatives and metabolites left isolated after the clustering were simply referred to as metabolites hereafter.

#### Multivariate analyses

Given the number of studied metabolites, penalized conditional logistic regression models were used to estimate mutually adjusted associations with cancer risk. Since body mass index (BMI) could be a strong confounder of the relationship between several of the examined metabolites [33, 34] and cancers [35–39], metabolite-specific linear models were used to compute residuals on BMI. To account for the large number of metabolites and leverage possible commonalities among the metabolic disorders preceding cancer development for different cancer types, estimation was based on the data-shared lasso [27–29], an extension of the lasso [40] allowing the analysis of case-control studies with multiple disease types. For each metabolite, the data-shared lasso decomposes its type-specific odds ratio as the product of (i) an overall odds ratio capturing the overall association with cancer and (ii) type-specific deviations from this overall odds ratio. Then, the method identifies whether its overall (mutually adjusted) association with cancer is null or not and also whether some of its type-specific associations deviate from its (possibly null) overall association with cancer. Compared to more standard approaches, the data-shared lasso was shown to perform particularly well for the identification of features with a consistent non-null association with multiple disease types, while also allowing for the identification of type-specific associations [29]. The data-shared lasso along with its implementation are described further in the Supplementary Material (Additional file 1: Section 2 [27–29, 41–45]).

To assess the robustness of the identified associations, the data-shared lasso was applied repeatedly on 100 bootstrap samples generated from the original sample [46]. Moreover, following the rationale of the lasso-OLS hybrid [47], associations identified by the data-shared lasso were further inspected using unpenalized conditional logistic regression models, (i) to quantify their strength and investigate possible heterogeneity among the type-specific associations beyond those identified by the data-shared lasso (see Additional file 1: Section 3 [47, 48] for details); (ii) to assess possible departure from linearity by comparing models with natural cubic splines to models with linear terms only; and (iii) to assess possible attenuation after excluding, in turn, first 2 and first 7 years of follow-up (to examine potential reverse causation and more generally assess the impact of time to diagnosis on our findings), after adjustment for additional factors (education level, waist circumference, height, physical activity, smoking status, alcohol intake, use of non-steroidal anti-inflammatory drugs, and, for women, menopausal status and phase of menstrual cycle in premenopausal women), and after reintegrating the pairs comprising at least one hormone user. Effect modification by BMI was assessed under standard (i.e., non-conditional) logistic regression models after breaking the matching and correcting metabolite measurements for batch and study effects [31]. Finally, to assess the impact of the exclusion of pairs with missing information on tumour stage in the prostate study, the data-shared lasso was applied to 100 bootstrap samples generated from the sample comprising all pairs from the prostate study, after considering an additional subtype (“unknown stage”) for prostate cancer.

#### Univariate analyses

For comparison, non-mutually adjusted associations with cancer risk were estimated for each metabolite in conditional logistic regression models adjusted for BMI. Those analysis and subsequent results are presented in the Supplementary Material (Additional file 1: Section 4 [49]).

#### Analysis of additional metabolites

The 16 metabolites (Additional file 2: Table S1) that were not acquired in the second colorectal cancer study (AbsoluteIDQ p150 kit) were not included in our main analysis and were examined in a reduced sample, using the methods described above.

## Results

### Data pre-processing

**Fig. 1:**
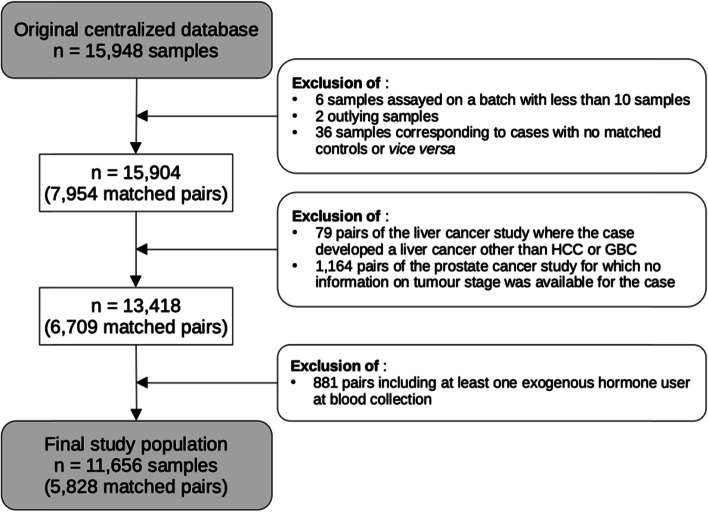
Flowchart summarizing the exclusion criteria to derive the final sample used in our main analysis. GBC stands for gallbladder and biliary tract cancer and HCC for hepatocellular carcinoma

Among the 118 metabolites that were measured in all cancer type-specific studies of the main analysis, the acylcarnitine C4-OH (C3-DC) was the only one that was missing in more than 25% of the samples of at least one study (prostate) and was excluded. Exclusions of subjects are detailed in Fig. 1. Briefly, 44 samples were initially excluded due to being either assayed on batches with less than 10 samples (6 samples), identified as outlying samples (2 samples), or unmatched to either a case or a control sample (36 samples). Seventy-nine pairs from the liver study were also excluded, having developed a liver cancer other than HCC or GBC, along with 1164 pairs from the prostate study for which no information on the tumour stage was available for the case. Finally, 881 pairs including at least one exogenous hormone user at blood collection were excluded.

### Description of the study population

A total of 11,656 EPIC participants were included in the analysis comprising 5828 matched case-control pairs. Cases were diagnosed at an average age of 64.4 years, 8.4 years after blood collection. The main characteristics of cases and controls in each study are displayed in Table 2.

**Table 2:** Main characteristics of the control (Ctrl) and case (Case) sub-populations in the eight cancer type-specific EPIC studies

	BrC study		CRC study		EnC study		KiC study		GBTC study		HCC study		Adv. PrC study		Loc. PrC study	
	***N*** = 1088 pairs		***N*** = 1500 pairs		***N*** = 689 pairs		***N*** = 511 pairs		***N*** = 85 pairs		***N*** = 121 pairs		***N*** = 533 pairs		***N*** = 1301 pairs	
	Ctrl	Case	Ctrl	Case	Ctrl	Case	Ctrl	Case	Ctrl	Case	Ctrl	Case	Ctrl	Case	Ctrl	Case
**Age at blood collection**																
Mean (SD)	51.8 (8.31)	51.8 (8.33)	57.0 (7.58)	57.1 (7.57)	54.3 (7.83)	54.3 (7.84)	55.8 (8.47)	55.8 (8.46)	58.7 (7.13)	58.7 (7.08)	59.9 (7.01)	59.9 (6.98)	57.6 (7.18)	57.6 (7.18)	57.9 (6.80)	58.0 (6.80)
**Age at cancer diagnosis**																
Mean (SD)	- -	60.4 (8.83)	- -	64.9 (8.18)	--	62.7 (8.16)	- -	64.5 (8.83)	- -	64.9 (7.60)	- -	66.1 (7.49)	--	66.3 (7.02)	--	67.1 (6.36)
**Sex**																
Female	1088 (100%)	1088 (100%)	769 (51.3%)	769 (51.3%)	689 (100%)	689 (100%)	197 (38.6%)	197 (38.6%)	48 (56.5%)	48 (56.5%)	35 (28.9%)	35 (28.9%)	0 (0%)	0 (0%)	0 (0%)	0 (0%)
**BMI (kg/m**^**2**^**)**																
Mean (SD)	25.7 (4.32)	26.2 (4.80)	26.5 (3.88)	27.2 (4.34)	26.0 (4.29)	28.2 (5.52)	26.7 (3.84)	27.8 (4.47)	26.9 (4.38)	27.3 (3.98)	26.9 (3.72)	28.4 (4.73)	26.7 (3.48)	27.0 (3.20)	27.5 (3.49)	27.2 (3.37)
**Education**																
None	56 (5.1%)	62 (5.7%)	124 (8.3%)	136 (9.1%)	67 (9.7%)	76 (11.0%)	42 (8.2%)	34 (6.7%)	6 (7.1%)	6 (7.1%)	6 (5.0%)	7 (5.8%)	28 (5.3%)	32 (6.0%)	106 (8.1%)	136 (10.5%)
Primary school completed	397 (36.5%)	377 (34.7%)	574 (38.3%)	526 (35.1%)	269 (39.0%)	231 (33.5%)	184 (36.0%)	186 (36.4%)	33 (38.8%)	39 (45.9%)	49 (40.5%)	52 (43.0%)	160 (30.0%)	166 (31.1%)	444 (34.1%)	411 (31.6%)
Technical/professional school	245 (22.5%)	254 (23.3%)	333 (22.2%)	334 (22.3%)	124 (18.0%)	118 (17.1%)	107 (20.9%)	109 (21.3%)	20 (23.5%)	15 (17.6%)	31 (25.6%)	38 (31.4%)	140 (26.3%)	127 (23.8%)	305 (23.4%)	306 (23.5%)
Secondary school	158 (14.5%)	178 (16.4%)	188 (12.5%)	227 (15.1%)	100 (14.5%)	127 (18.4%)	66 (12.9%)	72 (14.1%)	11 (12.9%)	8 (9.4%)	11 (9.1%)	5 (4.1%)	58 (10.9%)	59 (11.1%)	103 (7.9%)	91 (7.0%)
Longer education (incl. University deg.)	211 (19.4%)	195 (17.9%)	241 (16.1%)	227 (15.1%)	100 (14.5%)	100 (14.5%)	96 (18.8%)	93 (18.2%)	15 (17.6%)	17 (20.0%)	22 (18.2%)	17 (14.0%)	132 (24.8%)	124 (23.3%)	306 (23.5%)	324 (24.9%)
Not specified	21 (1.9%)	22 (2.0%)	40 (2.7%)	50 (3.3%)	29 (4.2%)	37 (5.4%)	16 (3.1%)	17 (3.3%)	0 (0%)	0 (0%)	2 (1.7%)	2 (1.7%)	15 (2.8%)	25 (4.7%)	37 (2.8%)	33 (2.5%)

The main analysis focused on 117 metabolites that were retained after the pre-processing step (Additional file 2: Table S1). As displayed in Additional file 2: Figure S1, strong positive correlations were observed between some metabolites, particularly between some of the glycerophospholipids (phosphatidylcholines, PCs, and lysophosphatidylcholines, lysoPCs) and sphingomyelins (SMs).

### Clustering of metabolites

**Fig. 2:**
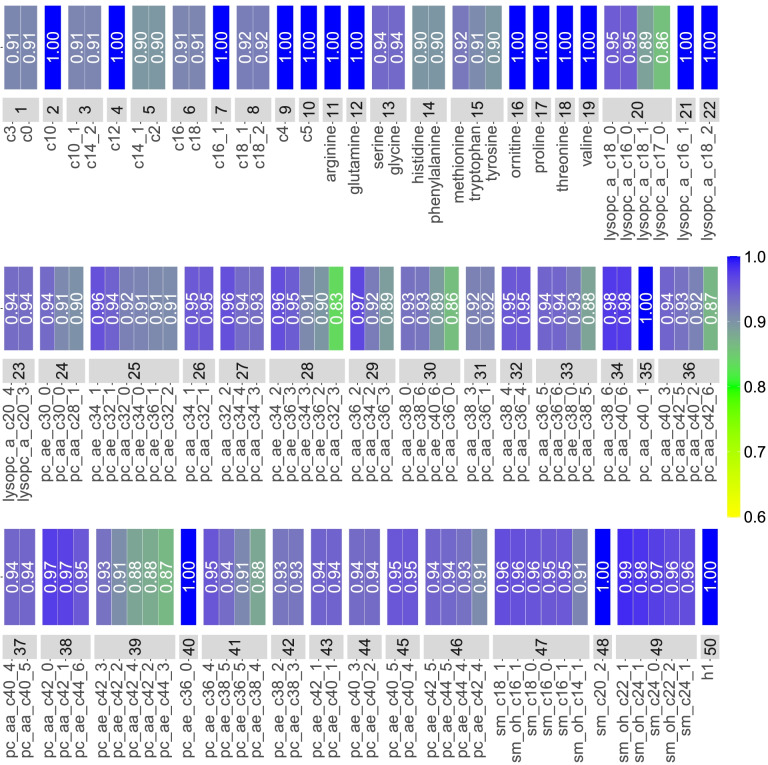
Description of the 50 “metabolites” retained for the main analysis. They include 33 clusters of strongly correlated metabolites and 17 “isolated” metabolites. For example, the 19th metabolite is an isolated metabolite (valine), while the 26th one is a cluster made of two phosphatidylcholines. For each cluster, correlations between its representative and the individual metabolites that compose that cluster are represented as a heat map (this correlation is 1 when the “cluster” is reduced to an isolated metabolite)

The hierarchical clustering applied to controls grouped 100 metabolites into 33 clusters of size ranging from 2 to 6 metabolites per cluster, while 17 metabolites remained isolated. As displayed in Fig. 2, clusters comprised metabolites of the same chemical class, and correlations between metabolites and their representative were consistently greater than 0.83. On average, clusters’ representatives explained 86% of the total variation of their cluster (range: 80–95%), and the 33 + 17 = 50 studied metabolites together explained more than 88% of the total variation of the original 117 metabolites.

### Multivariate analyses

**Fig. 3:**
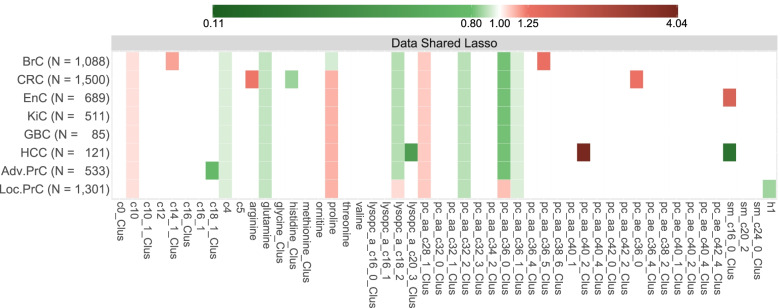
Summary of the main results from the multivariate pan-cancer analysis. It evaluated mutually adjusted associations between each feature (more precisely, their residuals after adjustment for BMI) and the risk of the eight cancer types, using a data-shared lasso penalty. White entries correspond to the absence of identified associations, while green and red entries correspond to inverse and positive associations, respectively. The more intense the colour, the larger the absolute value of the log-odds ratio (that were re-estimated in multivariate unpenalized conditional regression models; see Additional file 1: Section 3.a for details). The *x*-axis represents the 50 features (33 cluster representatives and 17 isolated metabolites). In the labels of the *y*-axis, numbers correspond to the numbers of pairs for each type-specific cancer, while BrC stands for breast cancer, CRC for colorectal cancer, EnC for endometrial cancer, KiC for kidney cancer, GBC for gallbladder and biliary tract cancer, HCC for hepatocellular carcinoma, and Adv.PrC and Loc.PrC for advanced and localized prostate cancers, respectively

**Fig. 4:**
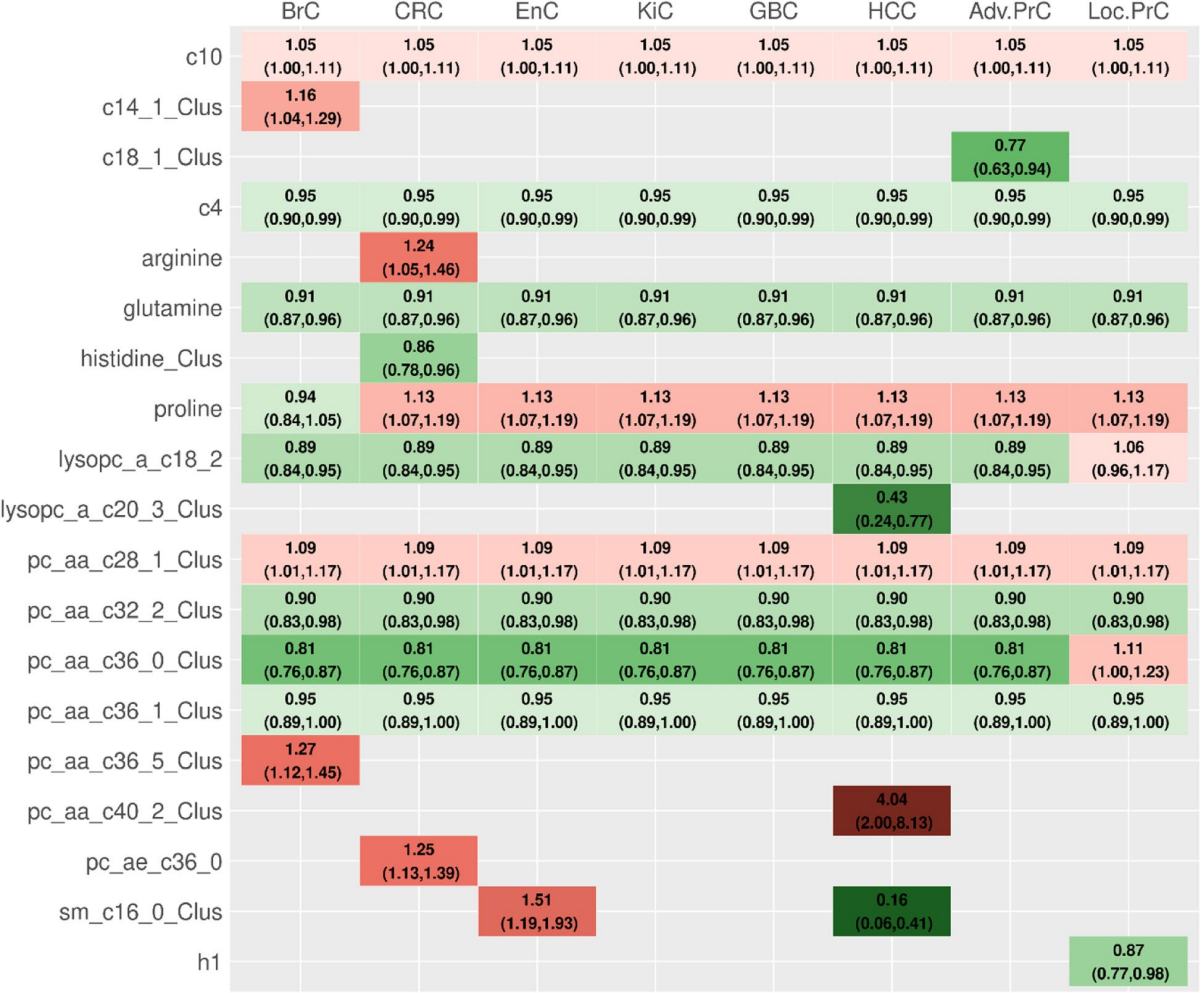
Summary of the mutually adjusted associations identified by the data-shared lasso. Only the 19 features (8 isolated metabolites and 11 cluster representatives) for which the data-shared lasso identified an association with at least one cancer type are presented on the *y-*axis. Point estimates and 95% confidence intervals of the corresponding odds ratios were obtained through non-penalized conditional logistic regression models using the design matrix derived from the positions of the non-zero components in the data-shared lasso vector estimate $\left(\hat{\boldsymbol{\mu}, \kern0.5em }{\hat{\boldsymbol{\delta}}}_1,\cdots, {\hat{\boldsymbol{\delta}}}_K\right)$; see Additional file 1: Section 3.a for details. They have to be interpreted with caution since they are the result of post-selection inference. In the labels of the columns, BrC stands for breast cancer, CRC for colorectal cancer, EnC for endometrial cancer, KiC for Kidney cancer, GBC for gallbladder and biliary tract cancer, HCC for hepatocellular carcinoma, and Adv.PrC and Loc.PrC for advanced and localized prostate cancers, respectively

As displayed in Figs. 3 and 4, the data-shared lasso identified nine metabolites with a non-null overall association with cancer: butyrylcarnitine (acylcarnitine C4), glutamine, lysoPC a C18:2, and three clusters of PCs (those containing PC aa C32:2, PC aa C36:0, and PC aa C36:1, respectively), with an inverse overall association with cancer risk, and decanoylcarnitine (acylcarnitine C10), proline, and the cluster of PCs that included PC aa C28:1 with a positive overall association. Cancer type-specific deviations from the overall association with cancer risk were identified for three of these metabolites: the association between proline and breast cancer risk was inverse or null, while the associations between lysoPC a C18:2 and the cluster containing PC aa C36:0 with localized prostate cancer were positive or null.

Several cancer type-specific associations were identified among the remaining 41 metabolites. Specifically, positive associations were observed between breast cancer risk and two clusters that included tetradecenoylcarnitine (acylcarnitine C14:1) and PC aa C36:5, respectively. The risk of colorectal cancer was positively associated with arginine and PC ae C36:0 and inversely associated with the cluster that included histidine. The risk of HCC was positively associated with the cluster containing PC aa C40:2 and inversely associated with the two clusters that included lysoPC a C20:3 and SM C16:0, respectively. This latter cluster was also positively associated with endometrial cancer risk. The cluster that included octadecenoylcarnitine (acylcarnitine C18:1) was inversely associated with the risk of advanced prostate cancer. Finally, the risk of localized prostate cancer was inversely associated with hexoses (H1).

The strength of the associations identified by the data-shared lasso was similar after excluding, in turn, the first 2 and the first 7 years of follow-up (Additional file 2: Fig. S2), and after reintegrating the 881 pairs comprising at least one hormone user (Additional file 2: Fig. S3). Likewise, models adjusted for additional factors produced similar associations (Additional file 2: Fig. S2), except for the overall association with cancer for the cluster that included PC aa C28:1, whose odds ratio (OR) was attenuated from 1.09 (95% confidence interval: 1.01–1.17) to 1.04 (0.98–1.12), and for the association between endometrial cancer risk and the cluster that included SM C16:0, whose OR decreased from 1.51 (1.19–1.93) to 1.20 (0.97–1.47). For each overall association and type-specific deviation identified by the data-shared lasso, linearity and absence of effect modification by BMI were compatible with our data (Additional file 2: Fig. S4). Focusing on the nine metabolites that had a non-null overall association with cancer, the analysis presented in Additional file 2: Fig. S5 suggested possible cancer type-specific deviations from the overall associations beyond the three ones identified by the data-shared lasso, in particular for HCC (with acylcarnitine C4, proline, and the cluster that comprises PC aa C36:1) and for kidney cancer (with acylcarnitines C10 and C4 and the cluster that comprises PC aa C36:1). However, none of the comparisons between the models identified by the data-shared lasso and the nine “extended” models used to derive these fully cancer type-specific associations reached statistical significance (Additional file 2: Fig. S5).

As displayed in Table 3 (third column), 15 out of the 22 associations identified by the data-shared lasso were replicated in more than 50% of the bootstrap samples. As displayed in Table 4, three inverse cancer type-specific associations that were not identified by the data-shared lasso on the original sample were identified in more than 55% of the bootstrap samples: the cluster comprising glycine with endometrial cancer risk (identified in 65% of the bootstrap samples) and the cluster containing decenoylcarnitine (acylcarnitine C10:1) with risk of kidney cancer (56%) and lysoPC a C16:1 with risk of localized prostate cancer (84%). Positive associations between arginine and kidney cancer risk (74%) and between the cluster containing lysoPC a C16:0 and localized prostate cancer risk (86%) were also observed in more than 55% of the bootstrap samples.

**Table 3:** Robustness of the associations identified in the main analysis. For each identified association, the proportion of bootstrap samples on which it was replicated is reported (in bold when ≥50%)

Feature	Cancer type^a^	Proportion of bootstrap samples^b^	Proportion of bootstrap samples^c^
**Overall associations with cancer risk**			
c10	Overall	**62%**	**59%**
c4	Overall	47%	39%
Glutamine	Overall	**73%**	**76%**
Proline	Overall	**65%**	**50%**
lysopc_a_c18_2	Overall	**57%**	47%
pc_aa_c28_1_Clus	Overall	**57%**	**64%**
pc_aa_c32_2_Clus	Overall	49%	**71%**
pc_aa_c36_0_Clus	Overall	**86%**	**95%**
pc_aa_c36_1_Clus	Overall	**50%**	40%
**Cancer type-specific associations**			
c14_1_Clus	BrC	**80%**	**76%**
Proline	BrC	**77%**	**70%**
pc_aa_c36_5_Clus	BrC	36%	47%
Arginine	CRC	**88%**	19%
his_Clus	CRC	**81%**	**72%**
pc_ae_c36_0	CRC	**80%**	46%
sm_c16_0_Clus	EnC	**85%**	**87%**
lysopc_a_c20_3_Clus	HCC	32%	47%
pc_aa_c40_2_Clus	HCC	**61%**	34%
sm_c16_0_Clus	HCC	**90%**	**78%**
c18_1_Clus	Adv.PrC	40%	49%
lysopc_a_c18_2	Loc.PrC	14%	23%
pc_aa_c36_0_Clus	Loc.PrC	49%	41%
h1	Loc.PrC	**75%**	**68%**

^a^BrC stands for breast cancer, CRC for colorectal cancer, EnC for endometrial cancer, HCC for hepatocellular carcinoma, and Adv. Prc and Loc.PrC for advanced and localized prostate cancers, respectively

^b^Bootstrap samples were generated from the original sample of 5828 matched case-control pairs with information on 117 metabolites (corresponding to 50 features after the clustering step)

^c^Bootstrap samples were generated from the sample which comprised 4761 matched case-control pairs with information on 133 metabolites (corresponding to 65 features after the clustering step) after excluding the participants of the second CRC study

**Table 4:** Other associations identified in a large proportion of the bootstrap samples. Associations identified in at least 55% of both bootstrap analyses are reported, along with the proportion of bootstrap samples in which they were identified, and the corresponding average log-odds ratio (as estimated by the data-shared lasso on each bootstrap sample)

Feature	Cancer type^a^	Proportion of bootstrap samples^**1**^	Average log-OR^b^	Proportion of bootstrap samples^**2**^	Average log-OR^c^
**Overall associations with cancer risk**					
Glutamate	Overall	--	--	55%	0.09
Spermine	Overall	--	--	78%	−0.10
**Type-specific associations**					
gly_Clus	EnC	65%	−0.17	78%	−0.14
c10_1_Clus	KiC	56%	−0.18	56%	−0.17
lysopc_a_c16_1	Loc.PrC	84%	−0.19	78%	−0.18
Arginine	KiC	74%	0.23	71%	0.21
lysopc_a_c16_0_Clus	Loc.PrC	86%	0.24	79%	0.22
Glutamate	BrC	--	--	56%	−0.14
Serotonin	CRC	--	--	84%	0.35

^a^BrC stands for breast cancer, CRC for colorectal cancer, EnC for endometrial cancer, KiC for kidney cancer, and Loc.PrC for localized prostate cancer

^b^Bootstrap samples were generated from the original sample of 5828 matched case-control pairs with information on 117 metabolites (corresponding to 50 features after the clustering step)

^c^Bootstrap samples were generated from the original sample which comprised 4761 matched case-control pairs with information on 133 metabolites (corresponding to 65 features after the clustering step) after excluding the participants of the second CRC study

Results obtained on the bootstrap samples generated from the extended sample comprising all the pairs from the prostate study are presented in Additional file 2: Tables S2 and S3. Fifteen associations out of the 22 identified in our main analysis were replicated in more than 50% of these bootstrap samples. A few additional overall and type-specific associations were identified in a large proportion of the bootstrap samples (see Additional file 2: Table S3). In particular, an inverse association between acylcarnitine C10 and unknown stage prostate cancer was observed in 80% of the samples.

### Univariate analysis

The results from the univariate analysis are presented in the Supplementary Material (Additional file 1: Section 4) and in Additional file 2: Fig. S6.

### Analysis of the extended list of metabolites

After excluding 2134 samples from the second colorectal cancer study which used a different platform that measured a lower number of metabolites, 16 additional metabolites could be evaluated (Additional file 2: Table S1). Among them, the clustering step grouped leucine and isoleucine together. The analysis of this extended list of metabolites then focused on 65 metabolites (31 isolated metabolites and 34 cluster representatives), measured in 9522 participants. As displayed in Table 3, 11 out of the 22 associations identified in the main analysis presented above were again replicated in more than 50% of the bootstrap samples generated from this reduced sample. Four associations that were not identified in our previous analyses were identified in more than 55% of these new bootstrap samples (Table 4): an overall positive association between cancer risk and glutamate (55% of the bootstrap samples), an overall inverse association between cancer risk with spermine (78%), and two cancer type-specific associations between glutamate with breast cancer risk (inverse, 56%) and between serotonin and colorectal cancer risk (positive, 84%).

## Discussion

Using available metabolomics data from eight cancer-specific matched case-control studies nested within the EPIC cohort, we investigated the relationship between pre-diagnostic blood levels of over one hundred metabolites and risks of breast cancer, colorectal cancer, endometrial cancer, gallbladder and biliary tract cancer, HCC, kidney cancer, and localized and advanced prostate cancers. In our main analysis, we found nine metabolites associated with cancer risk across different cancer types, suggesting the existence of shared metabolic pathways, as well as fourteen cancer type-specific associations. These identified associations were found to be robust after extensive sensitivity analyses: in particular, they were not attenuated after exclusion of the first years of follow-up, hence were less likely to be due to reverse causality, were not attenuated after adjustment for relevant cancer risk factors, were not modified by BMI, and did not deviate significantly from linearity. In additional analyses, in particular those based on bootstrap samples, we identified several additional metabolites possibly associated with the risk of specific cancer types or with cancer risk across different cancer types.

Our results suggested that concentrations of glycerophospholipids (phosphatidylcholines and lysophosphatidylcholines) could be linked to the risk of cancer overall as well as to specific cancer types. The role of glycerophospholipids in carcinogenesis is not fully understood but could be related to their documented anti-inflammatory properties, protection from oxidative stress, inhibition of cell proliferation, and induction of apoptosis [50–52]. We observed a consistent inverse association between cancer risk with lysoPC a C18:2 as well as three clusters of phosphatidylcholines across all studied cancer types, except localized prostate cancer for which the association with lysoPC a C18:2 and one cluster of phosphatidylcholines was absent, or positive. An inverse association was previously reported between lysoPC a C18:2 with T2D in different studies [7, 53] as well as with risks of breast, colorectal, and prostate cancers in the pan-cancer analysis conducted in the EPIC Heidelberg study [26]. Our results regarding the three clusters of phosphatidylcholines were in line with many previously reported inverse associations between cancer and phosphatidylcholines [11, 12, 15, 16, 20, 54]. Besides, we identified a positive association between the cluster that included PC aa C28:1 and cancer risk across all studied cancer types. This cluster also comprised PC ae C30:0, for which a positive association was reported with risks of breast, colorectal, and prostate cancers in the EPIC Heidelberg study [26]. Cancer type-specific positive associations were found for the cluster containing PC aa C36:5 with breast cancer, PC ae C36:0 with colorectal cancer, and the cluster containing PC aa C40:2 with HCC. These three clusters were correlated with one another (Pearson correlation greater than 0.48), indicating that higher levels of these phosphatidylcholines might contribute to the development of these three cancer types.

We also observed robust associations between specific circulating amino acids and cancer risk. Our results suggested that proline was positively related to cancer risk across all studied cancer types, except breast cancer and possibly HCC (see Additional file 2: Fig. S5). A positive association between proline and prostate cancer risk was previously reported in EPIC [12]. In addition, a drosophila model of high-sugar diet [55] recently highlighted the possible role of proline in tumour growth, and proline was also found to distinguish colorectal cancer patients from those with adenomas [56] and to be associated with metastasis formation [57]. In the body, proline is generally synthesized via the glutamate/pyrroline 5-carboxylate pathway [58]. Glutamate was also found to be positively related to the risk of all cancer types except for breast cancer in our analysis. Moreover, glutamate is formed from the degradation of glutamine, which was inversely associated with overall cancer risk. Although prior studies of the French E3N and SU.VI.MAX cohorts reported a positive association between glutamine and premenopausal breast cancer [59, 60], our results regarding glutamine and glutamate were consistent with those of many previous studies that reported inverse associations between glutamine and risk of colorectal cancer [18], HCC [19, 61], and T2D [7, 25] and positive associations between glutamate and risk of premenopausal breast cancer [60], kidney cancer [15], HCC [19, 61], and T2D [7]. Lower serum levels of glutamine were also observed in kidney cancer [62] and ovarian cancer [63] cases compared to controls. Glutamine is an energy substrate for cancer cells and makes a major contribution to nitrogen metabolism. Alterations in glutamine-glutamate equilibrium often reflect energetic processes related to cancer metabolism [64]. It is possible that altered levels of glutamine and glutamate in individuals subsequently diagnosed with cancer may reflect ongoing metabolic processes related to cancer development and as such may serve as an early biomarker of cancer risk. However, the inverse association between glutamine levels and overall cancer risk observed in our analysis was only slightly attenuated after excluding, in turn, the first 2 and the first 7 years of follow-up suggesting that changes in the glutamine-glutamate may precede cancer development.

Our analysis additionally identified two positive and two inverse cancer type-specific associations with circulating amino acids. We observed an inverse association between colorectal risk and the cluster containing histidine, for which previous studies reported inverse associations with risks of colorectal cancer and T2D [25], while a positive association was reported with breast cancer [60]. Also, lower serum levels of histidine were previously reported in ovarian cancer cases compared to controls [65]. Our results further suggested an inverse association between endometrial cancer risk and the cluster composed of glycine and serine, in line with previous results from the EPIC cancer-specific study of endometrial cancer [14]. Previous studies also reported inverse associations between glycine and/or serine with risks of T2D [25]. Finally, our analysis suggested a positive association between arginine with risks of colorectal and kidney cancers (Table 4). Arginine plays a key role in nitric oxide production and polyamines synthesis [66]. Both have been found to be associated with tumour growth, with polyamines enhancing it and nitric oxide inhibiting it. Arginine’s influence on tumour growth thus might be related to the relative activity of those two pathways. For instance, arginine was previously found to be positively associated with breast cancer in the E3N cohort [60], while an inverse association with breast cancer was reported in EPIC [11].

Regarding the biogenic amines, we found a positive association between serotonin levels and colorectal cancer risk, consistent with previous results from the CORSA case-control study and a previous EPIC analysis of colon cancer [67]. We also found a consistent inverse association between spermine and the risk of the eight studied cancer types. Like other polyamines, spermine is involved in cell proliferation and differentiation and has antioxidant properties [68], and dysregulation of polyamine metabolism is characteristic of multiple types of tumours [69]. It was previously reported that polyamine supplementation, in particular spermidine, which acts as an intermediate in the conversion of putrescine to spermine, could be related to reduced overall and cancer-specific mortality [70–72].

In our analysis, localized and advanced prostate cancers were considered as two different outcomes as previous results suggested that metabolic dysregulation might be predictive of advanced or aggressive prostate cancers only [12]. In fact, we observed some differences between the metabolites associated with risks of localized and advanced prostate cancers, respectively. Specifically, and as previously reported [12, 13], our results suggested that hexoses, glycerophospholipids, octadecenoylcarnitine (acylcarnitine C18:1), and/or octadecadienylcarnitine (acylcarnitine C18:2) could help differentiate the respective mechanisms involved in the development of aggressive and localized prostate tumours. On the other hand, the positive association with decanoylcarnitine (acylcarnitine C10), which was observed with risk of all cancer types, and in particular with both localized and advanced prostate cancer risk, was notably attenuated when including the unknown stage prostate cancer pairs: it was only detected in 44% of the bootstrap samples generated from that extended sample (see Additional file 2: Table S2), in line with the inverse association between decanoylcarnitine and unknown stage prostate cancer that was observed in 80% of the samples (Additional file 2: Table S3). Overall, these results suggested that the positive association between decanoylcarnitine and prostate cancer identified in our main analysis might not be real and might be due to an association between decanoylcarnitine and cancer stage missingness in our prostate cancer study.

Some metabolites identified in our study were previously associated with established cancer risk factors, such as obesity [33, 34]. In particular, a recent metabolomics study of BMI reported inverse associations with glutamine, lysophosphatidylcholine a C18:2, and phosphatidylcholine PC aa C38:0 (which was clustered with PC aa C36:0 in our analysis) and a positive association with glutamate. Directions of the associations with BMI were consistent with those identified in our study with cancer risk after adjustment for BMI, indicating that these metabolites might be mediators of the obesity-cancer relationship.

Our study has several strengths. First, it relied on a large sample of pre-diagnostic metabolomics data acquired among 5828 case-control pairs in nested studies on eight cancer types within a large prospective cohort, on average 6.4 years before cases developed cancer. Second, in a context where some metabolites might be predictive of cancer risk for multiple cancer types, the data-shared lasso used in our analysis automatically accounted for or ignored cancer types when assessing the association between each metabolic feature with cancer risk, depending on whether heterogeneity among the cancer type-specific associations was supported by the data for that particular feature. The comparison of results produced by the standard univariate analyses and the data-shared lasso illustrated the interest of the latter. First, the data-shared lasso benefited from the increased statistical power of the pooled analysis for the identification of metabolites that could be involved in cancer development for multiple cancer types: for example, butyrylcarnitine (acylcarnitine C4) was not associated with cancer risk in any of the cancer type-specific univariate analyses, while it was in the univariate pooled analysis and in the data-shared lasso analysis. Moreover, unlike the simple pooled analysis, the data-shared lasso would not necessarily mask cancer type-specific associations: for example, the data-shared lasso identified a positive association between the cluster containing tetradecenoylcarnitine (acylcarnitine C14:1) and breast cancer risk, as the univariate analysis of the breast cancer study did, while the univariate pooled analysis could not. Another key difference between the standard univariate analyses and the data-shared lasso is that the latter allowed the investigation of mutually adjusted associations, hence the identification of metabolites or clusters of metabolites whose association with cancer risk could not be explained away by other metabolites included in our analysis. Furthermore, mutual adjustment revealed associations that could not be detected in minimally adjusted models, such as the one between arginine and colorectal cancer risk, which was not apparent in models not adjusted for glutamine and histidine. Another strength of our study stemmed from the extensive sensitivity analyses that we carried out.

On the other hand, identifying cancer risk factors is particularly challenging when candidate risk factors are strongly correlated with one another. Here, we clustered the most strongly correlated metabolites together prior to applying the data-shared lasso. As a sensitivity analysis, the data-shared lasso was applied to the original set of 117 metabolites, thus ignoring the clustering step, and the results were largely consistent with those of our main analysis (Additional file 2: Fig. S7). Moreover, because strong correlations remained among some of the metabolites produced by the hierarchical clustering (Additional file 2: Fig. S8, Additional file 2: Fig. S9), we applied the data-shared lasso to multiple bootstrap samples to gauge the robustness and specificity of the associations identified in our main analysis. Although most of the identified associations were replicated in a large proportion of bootstrap samples, a few of them were less robust, hence more questionable. For example, the identified inverse association between HCC risk and the cluster that included lysoPC a C20:3 was replicated in 32% of the bootstrap samples only. This lack of robustness could be due to the strong correlation between this cluster and the other three studied metabolites related to lysoPCs (Pearson correlation greater than 0.65; Additional file 2: Fig. S8). As a matter of fact, an inverse association between HCC risk and at least one of the four metabolites related to lysoPCs was identified in 78% of the bootstrap samples. Overall, these results were suggestive of a stronger inverse association with features related to lysoPCs for HCC compared to the other cancer types, but our analysis failed to unambiguously identify which specific lysoPCs might underlie this stronger inverse association. An additional limitation for interpreting the lipid results is the lack of specificity for lipids measured with the AbsoluteIDQ p180/p150 kits as a result of the FIA method [73, 74], which does not allow for unambiguous identification of the compounds measured since the signal observed could correspond to several compounds. Moreover, the limited sample size for some of the studied cancer types (in particular, gallbladder and biliary tract cancer and HCC) was a limitation for the identification of cancer type-specific deviations. In this respect, we complemented our analysis by the inspection of estimates computed under models derived from the one identified by the data-shared lasso but that further allowed fully type-specific associations (Additional file 2: Fig. S5). Another potential limitation of our study was the lack of repeated measurements, yet previous studies suggested that blood levels of metabolites were relatively stable and that a single measurement might be sufficient to capture medium-term exposure [75–77].

## Conclusions

Our results confirmed the complex link between metabolism and cancer risk and highlighted the potential of metabolomics to identify possible informative markers associated with cancer risk and to gain insights into the biological mechanisms leading to cancer development. Our study indicated that specific metabolite families might be related to the risk of multiple cancer types. Some of these metabolites could reflect biological mechanisms underlying the carcinogenic effects of some established cancer risk factors, including obesity.

## Supplementary Information

**Additional file 1.** Supplementary material regarding (i) the definition of cancer cases for HCC, GBC, Adv.PrC and Loc.PrC; (ii) the definition and implementation of the data-shared lasso; (iii) the models used to derive point estimates and confidence intervals from the model selected by the data-shared lasso; and (iv) the univariate analysis conducted for comparison.

**Additional file 2: Supplementary tables and figures.** **Figure S1.** Pearson correlation between the 117 original metabolites. **Figure S2.** Sensitivity analyses of mutually adjusted ORs for the overall associations and cancer type-specific deviations. **Figure S3.** Sensitivity analysis of mutually adjusted ORs for the overall associations and cancer type-specific deviations with or without excluding hormone users. **Figure S4.** *p*-values of tests for departure from linearity and effect modification by BMI. **Figure S5.** ORs for the overall associations identified by the data-shared lasso with (i) the original model (ii) the extended type-specific model. **Figure S6.** Results from the univariate analyses. **Figure S7.** Comparison of the associations identified by the data-shared lasso when working with the 50 features (as in our main analysis) or with the original 117 metabolites. **Figure S8.** Pearson correlation between the 50 clusters. **Figure S9.** Pearson correlation between the 19 features related to at least one cancer site in our main analysis. **Table S1.** list of the 117 metabolites studied in the main analysis, and of the 16 additional metabolites studied when excluding the second colorectal study. **Table S2.** Robustness of the associations identified in the main analysis when including all the pairs from the prostate cancer study. **Table S3.** Other associations identified in a large proportion of bootstrap samples when including all the pairs from the prostate cancer study.

## Data Availability

The R scripts developed to implement the analyses will be made available on the GitHub platform, for easy access to all interested scientists. The EPIC data is not publicly available, but access requests can be submitted to the Steering Committee (https://epic.iarc.fr/access/submit_appl_access.php).

## Abbreviations

Adv.PrC: Advanced prostate cancer
BMI: Body mass index
BrC: Breast cancer
CRC: Colorectal cancer
CVD: Cardiovascular diseases
EnC: Endometrial cancer
EPIC: European Prospective Investigation into Cancer and Nutrition
FDR: False discovery rate
FIA: Flow injection analysis
GBC: Gallbladder and biliary tract cancer
HCC: Hepatocellular carcinoma
HZM: Helmholtz Zentrum
IARC: International Agency for Research on Cancer
ICL: Imperial College London
KiC: Kidney cancer
Lasso: Least absolute shrinkage and selection operator
LC: Liquid chromatography
LLOQ: Lower limit of quantification
Loc.PrC: Localized prostate cancer
LOD: Limit of detection
lysoPC: Lysophosphatidylcholine
MS/MS: Tandem mass spectrometry
OLS: Ordinary least square regression
OR: Odds ratio
PC: Phosphatidylcholine
PCA: Principal component analysis
PrC: Prostate cancer
SM: Sphingomyelin
T2D: Type 2 diabetes
ULOQ: Upper limit of quantification
