# Supplementary material for: Pan-cancer analysis of pre-diagnostic blood metabolite concentrations in the European Prospective Investigation into Cancer and Nutrition
Source: BMC Med. 2022 Oct 19;20:351. doi: 10.1186/s12916-022-02553-4 (PMC9580145; doi:10.1186/s12916-022-02553-4)
Supplement: Supplementary file 2 — Additional file 2: Supplementary tables and figures. Figure S1. Pearson correlation between the 117 original metabolites. Figure S2. Sensitivity analyses of mutually adjusted ORs for the overall associations and cancer type-specific deviations. Figure S3. Sensitivity analysis of mutually adjusted ORs for the overall associations and cancer type-specific deviations with or without excluding hormone users. Figure S4. p-values of tests for departure from linearity and effect modification by BMI. Figure S5. ORs for the overall associations identified by the data-shared lasso with (i) the original model (ii) the extended type-specific model. Figure S6. Results from the univariate analyses. Figure S7. Comparison of the associations identified by the data-shared lasso when working with the 50 features (as in our main analysis) or with the original 117 metabolites. Figure S8. Pearson correlation between the 50 clusters. Figure S9. Pearson correlation between the 19 features related to at least one cancer site in our main analysis. Table S1. list of the 117 metabolites studied in the main analysis, and of the 16 additional metabolites studied when excluding the second colorectal study. Table S2. Robustness of the associations identified in the main analysis when including all the pairs from the prostate cancer study. Table S3. Other associations identified in a large proportion of bootstrap samples when including all the pairs from the prostate cancer study. [file 12916_2022_2553_MOESM2_ESM.docx]

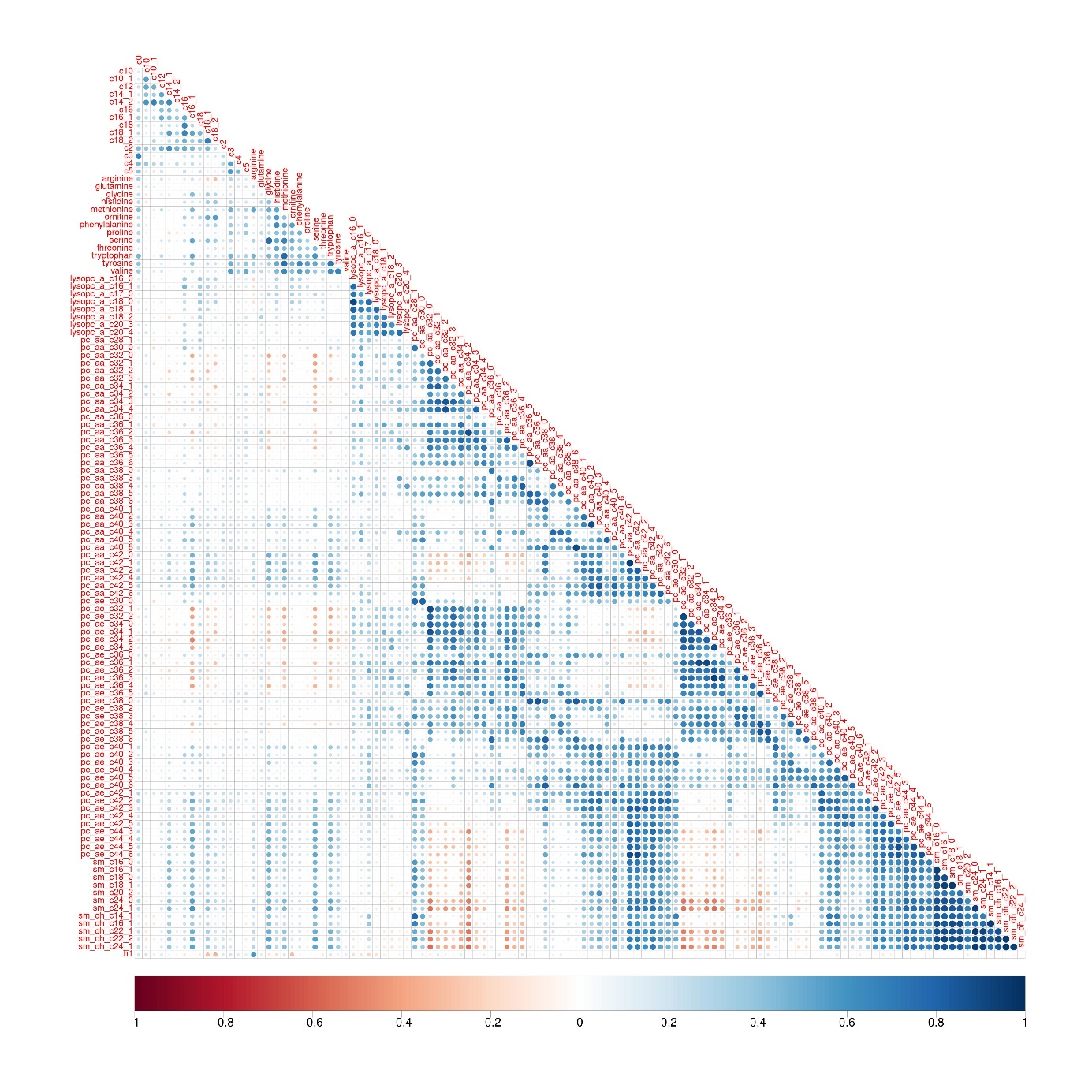
**Figure S1.** Pearson correlation between the 117 metabolites computed in the 5,985 controls of the eight cancer type-specific EPIC studies.

**
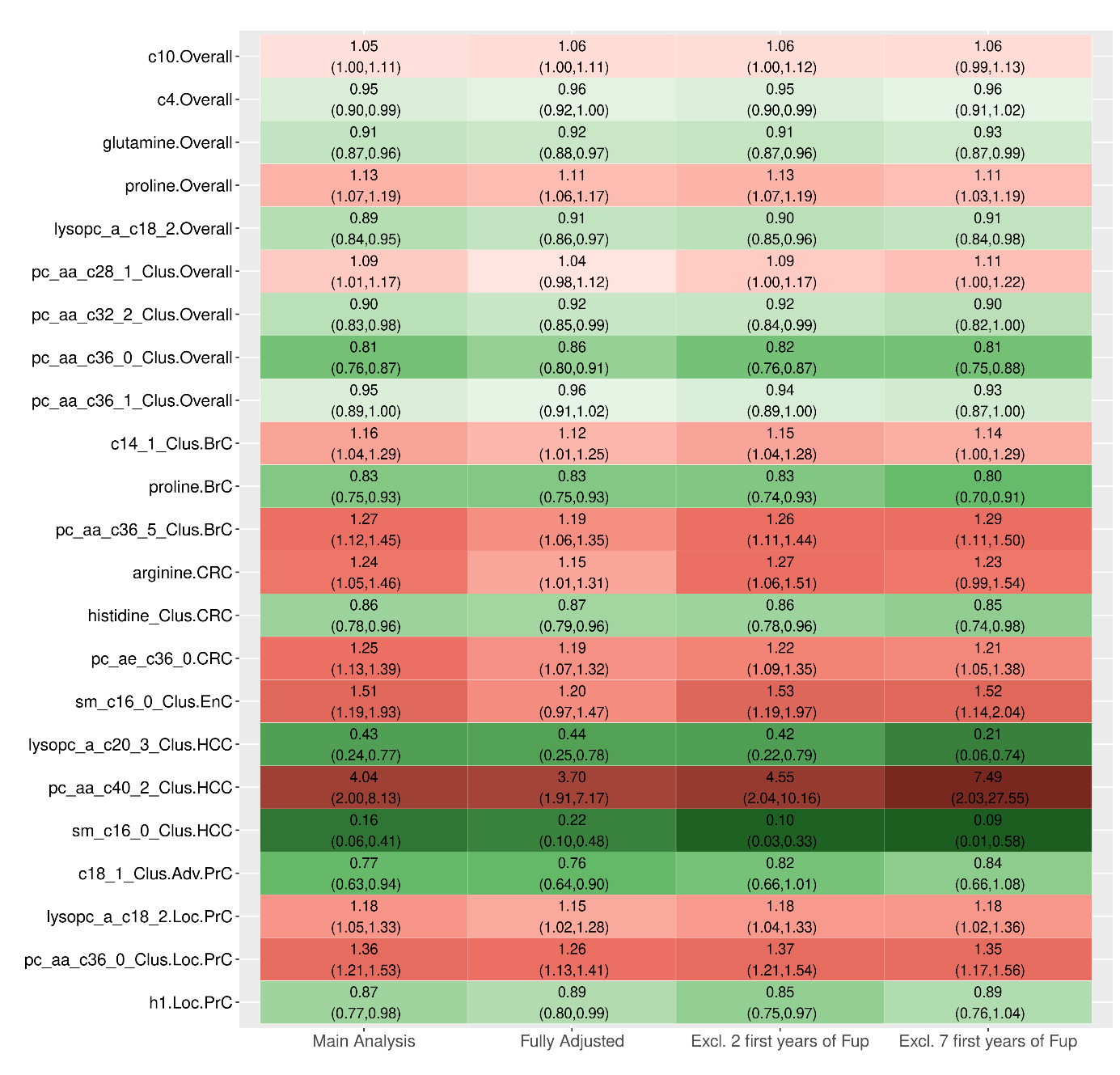
**

**Figure S2.** Sensitivity analyses of the mutually adjusted odd-ratios for the overall associations and cancer type-specific deviations around the overall association identified by the data shared lasso. Point estimates and confidence intervals were obtained through non-penalized conditional logistic regression models using the design matrix derived from the positions of the non-zero components in the data shared lasso vector estimate $\left( \hat{\boldsymbol{\mu},}{\hat{\boldsymbol{\delta}}}_{1}, \cdots{,\hat{\boldsymbol{\delta}}}_{K} \right)$, and based on: *(first column)* residuals of the metabolite measurements after adjustment for BMI (as for the data shared lasso); *(second column)* residuals of the metabolite measurements after adjustment for BMI, education level, waist circumference, height, physical activity, smoking status, alcohol intake, use of non-steroidal anti-inflammatory drugs, and, for women, menopausal status and phase of menstrual cycle; (*third column*) residuals of the metabolite measurements after adjustment for BMI , and excluding pairs for which the case developed cancer within the first two years of follow-up; and (*fourth column*) residuals of the metabolite measurements after adjustment for BMI, and excluding pairs for which the case developed cancer within the first seven years of follow-up. Point estimates and confidence intervals have to be interpreted with caution since they are the result of post-selection inference. In the labels of the y-axis, BrC stands for breast cancer, CRC for colorectal cancer, EnC for endometrial cancer, HCC for hepatocellular carcinoma, and Adv.PrC and Loc.PrC for advanced and localized prostate cancers, respectively.

**
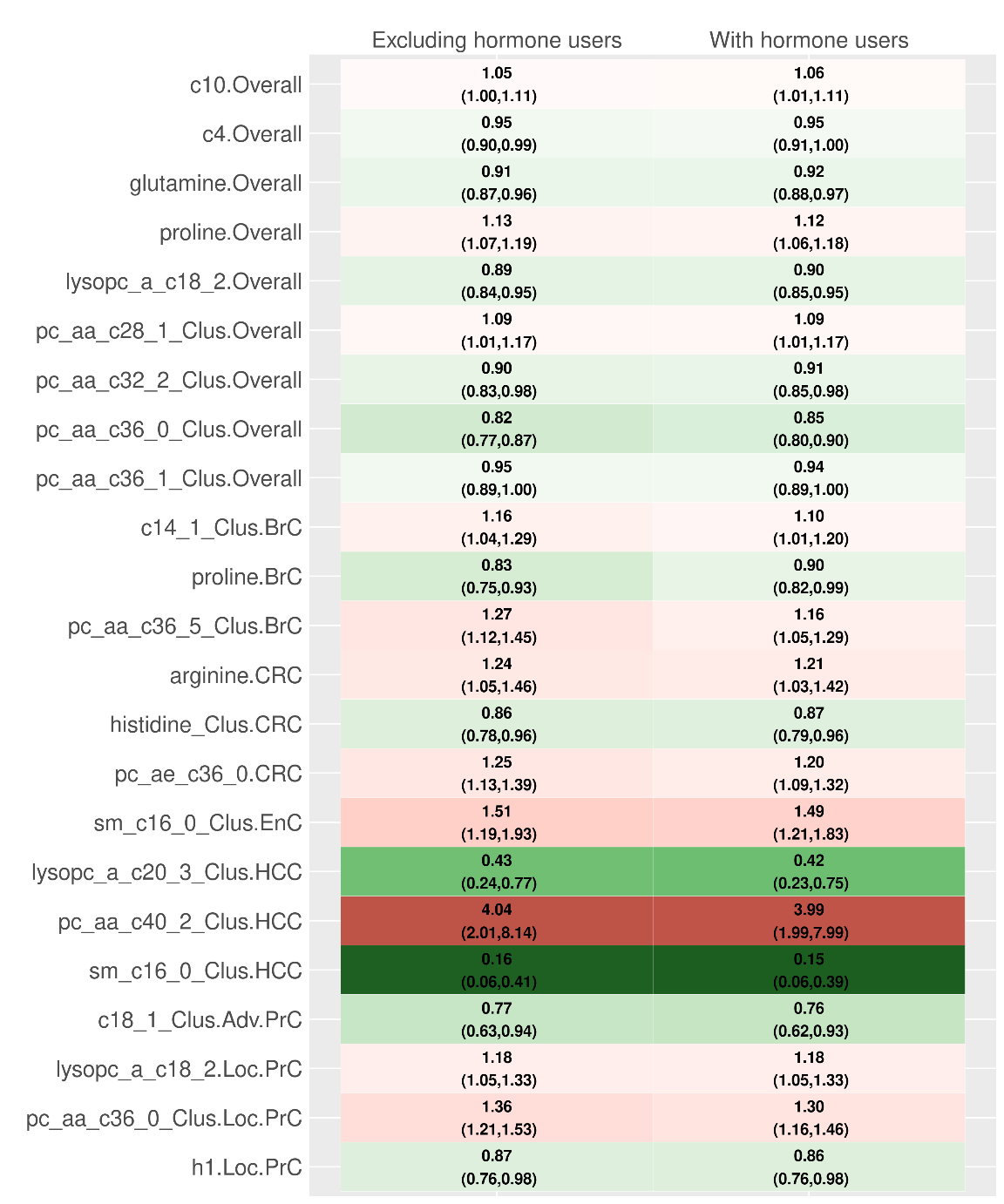
**

**Figure S3.** Mutually adjusted odd-ratios for the overall associations and cancer type-specific deviations around the overall association identified by the data shared lasso, with or without exclusion of the hormone users. Point estimates and confidence intervals were obtained through non-penalized conditional logistic regression models using the design matrix derived from the positions of the non-zero components in the data shared lasso vector estimate $\left( \hat{\boldsymbol{\mu},}{\hat{\boldsymbol{\delta}}}_{1}, \cdots{,\hat{\boldsymbol{\delta}}}_{K} \right)$, and based on: *(first column)* the main sample from which 881 pairs comprising at least one hormone user were excluded *(second column)* the extended sample comprising 6,709 pairs of individuals, including hormone users. Point estimates and confidence intervals have to be interpreted with caution since they are the result of post-selection inference. In the labels of the y-axis, BrC stands for breast cancer, CRC for colorectal cancer, EnC for endometrial cancer, HCC for hepatocellular carcinoma, and Adv.PrC and Loc.PrC for advanced and localized prostate cancers, respectively.


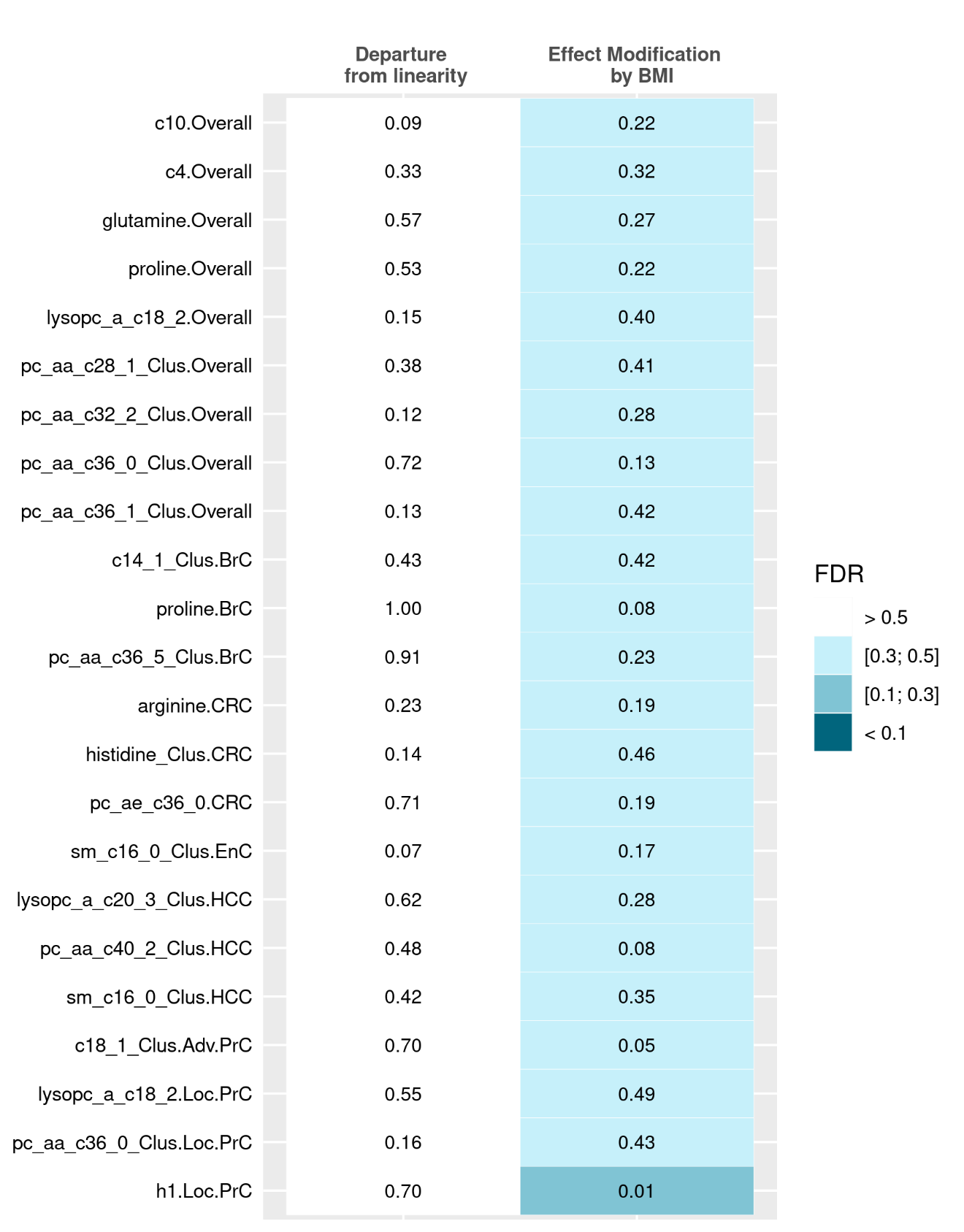


**Figure S4.** P-values of the statistical tests computed to assess *(i)* possible departure from linearity for the associations identified by the data shared lasso; and *(ii)* possible effect modifications by body mass index (BMI) for the associations identified by the data shared lasso. The background colour indicates the value of the corresponding FDR: in particular, none of the FDRs was below 0.10 (the lowest FDR was 0.27). P-values, and FDR, have to be interpreted with caution since they are the result of post-selection inference. In the labels of the y-axis, BrC stands for breast cancer, CRC for colorectal cancer, EnC for endometrial cancer, HCC for hepatocellular carcinoma, and Adv.PrC and Loc.PrC for advanced and localized prostate cancers, respectively.


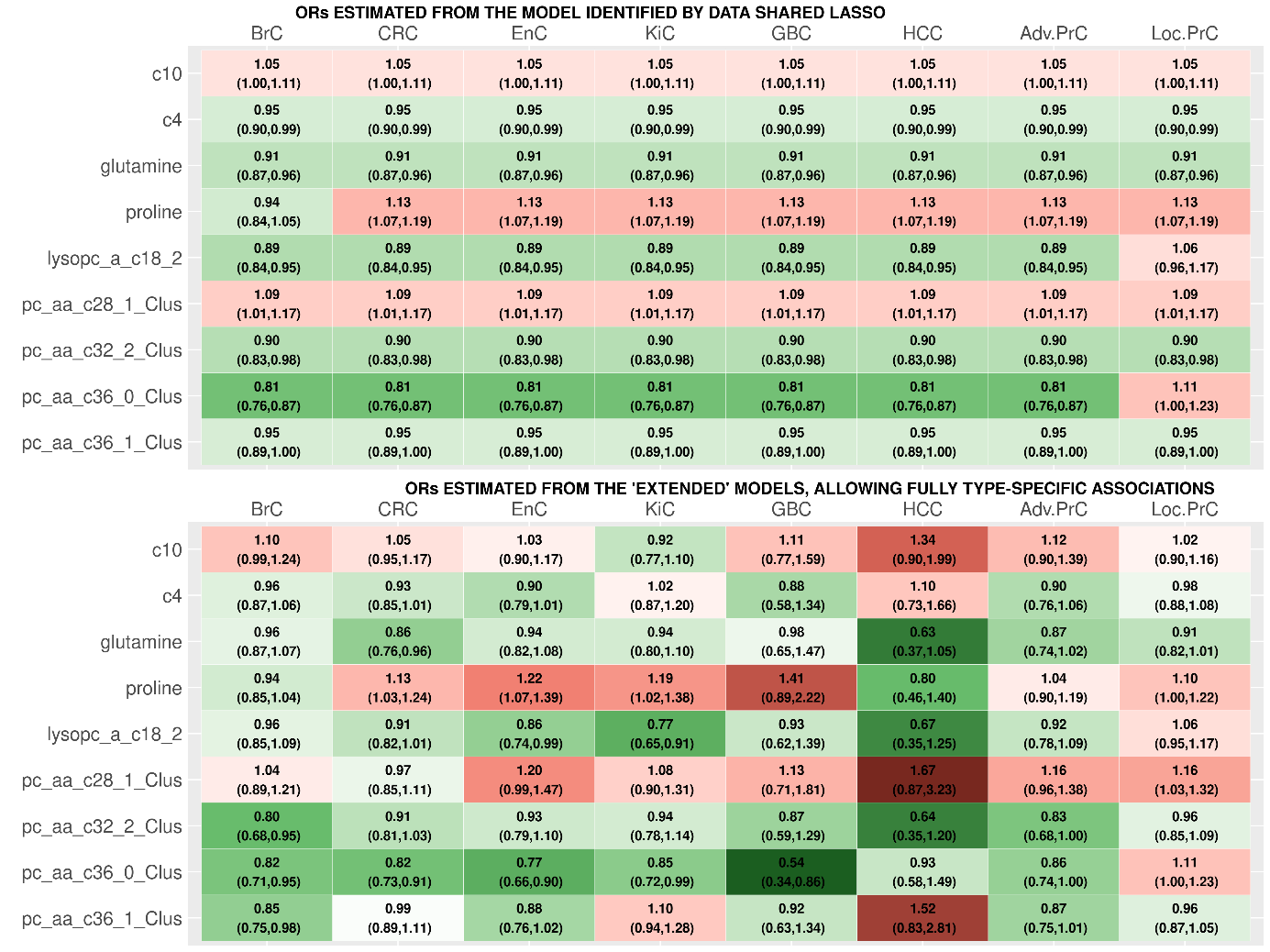


**Figure S5.** Association (odds-ratio) with risks of the eight cancer types for the nine metabolites that had an overall association with cancer risk in our main analysis. (*Top)* Point estimates and 95% confidence intervals were obtained through non-penalized conditional logistic regression models using the design matrix derived from the positions of the non-zero components in the data shared lasso vector estimate $\left( \hat{\boldsymbol{\mu},}{\hat{\boldsymbol{\delta}}}_{1}, \cdots{,\hat{\boldsymbol{\delta}}}_{K} \right)$; see Section 3.a) in the Supplementary Material for details. (*Bottom)* For each of the nine features, point estimates and 95% confidence intervals were obtained through non-penalized conditional logistic regression models using the design matrix derived from the positions of the non-zero components in the data shared lasso vector estimate $\left( \hat{\boldsymbol{\mu},}{\hat{\boldsymbol{\delta}}}_{1}, \cdots{,\hat{\boldsymbol{\delta}}}_{K} \right)$, but further allowing for fully type-specific associations between that particular feature and cancer risk; see Section 3.b) in in the Supplementary Material for details. The p-values of the likelihood ratio tests comparing the two types of models were 0.65, 0.85, 0.63, 0.42, 0.39, 0.32, 0.53, 0.55 and 0.11 for c10, c4, gln, pro, lysopc_a_c18_2, pc_aa_c28_1_Clus, pc_aa_c32_2_Clus, pc_aa_c36_0_Clus and pc_aa_c36_1_Clus, respectively, suggesting the absence of type-specific deviations beyond those identified by the data shared lasso for these nine metabolites. Point estimates, confidence intervals and p-values have to be interpreted with caution since they are the result of post-selection inference. In the column labels, BrC stands for breast cancer, CRC for colorectal cancer, EnC for endometrial cancer, KiC for Kidney cancer, GBC for gallbladder and biliary tract cancer, HCC for hepatocellular carcinoma, and Adv.PrC and Loc.PrC for advanced and localized prostate cancers, respectively.


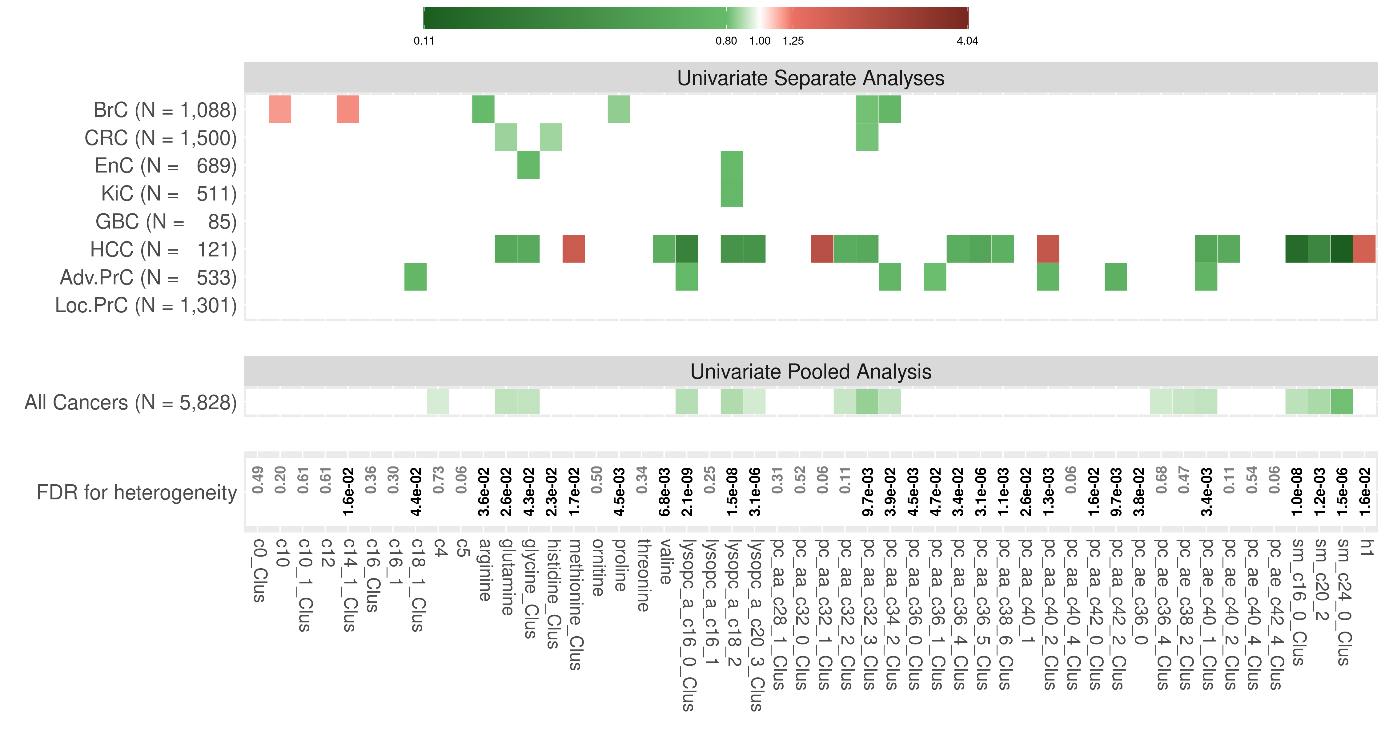
**Figure S6.** Summary of the results from our univariate analyses: *(top)* associations with an FDR < 5% in the univariate analyses of the association between each feature and risk of each cancer type in conditional logistic regression models adjusted for BMI; *(center)* associations with an FDR < 5% in the univariate analyses of the association between each feature and the risk of cancer (after pooling the data from the eight cancer-type specific studies together, thus ignoring cancer types), in conditional logistic regression models adjusted for BMI; *(bottom)* FDR for the statistical test of a heterogeneity among its (non-mutually adjusted) type-specific associations. In the top and center panels, white entries correspond to the absence of identified associations, while green and red entries correspond to inverse and positive associations, respectively. The more intense the colour, the larger the absolute value of the log-odds-ratio). The x-axis represents the 50 features (33 cluster representatives and 17 isolated metabolites). In the labels of the y-axis, numbers correspond to numbers of pairs for each type-specific cancer (and in total for the pooled analysis), while BrC stands for breast cancer, CRC for colorectal cancer, EnC for endometrial cancer, KiC for Kidney cancer, GBC for gallbladder and biliary tract cancer, HCC for hepatocellular carcinoma, and Adv.PrC and Loc.PrC for advanced and localized prostate cancers, respectively.


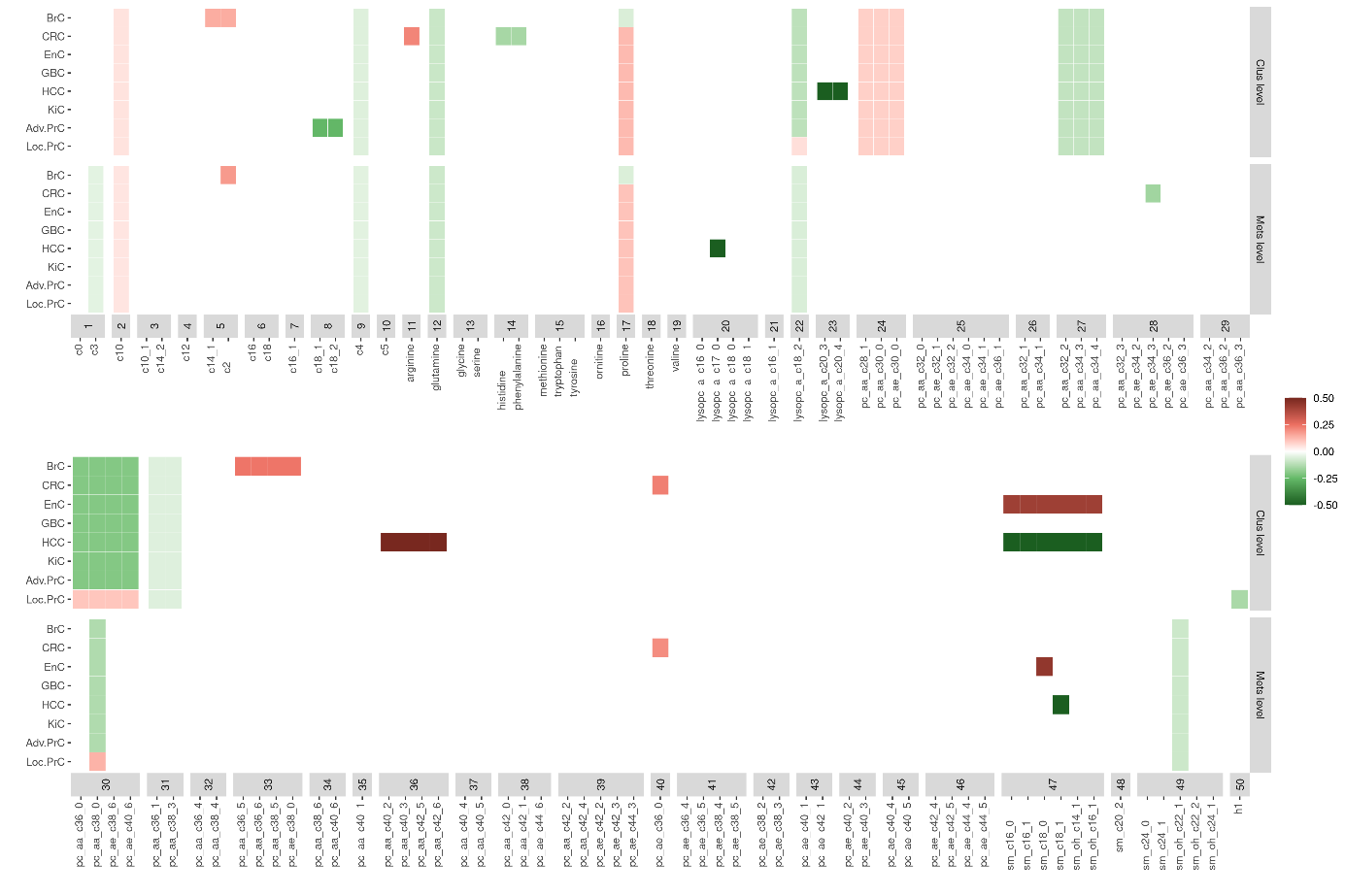


**Figure S7.** Comparison of the associations identified by the data shared lasso when working with the 50 features (Clus Level, as in our main analysis) or with the original 117 metabolites (Mets Level). The 117 metabolites are organized by cluster, and for the associations identified when working at the cluster level, the figure displays this association for all the metabolites that compose that cluster. Overall, the analysis conducted at the cluster level identified more associations, but most results from the two types of analyses were consistent. For lysoPCs for example, the two analyses did not identify exactly the same associations, but they both suggested *(i)* an overall inverse association with cancer risk and *(ii)* a stronger inverse association with HCC (the analysis conducted at the cluster level also suggested a weaker association with localized PrC). In the labels of the *y* axis, BrC stands for breast cancer, CRC for colorectal cancer, EnC for endometrial cancer, KiC for Kidney cancer, GBC for gallbladder and biliary tract cancer, HCC for hepatocellular carcinoma, and Adv.PrC and Loc.PrC for advanced and localized prostate cancers, respectively.


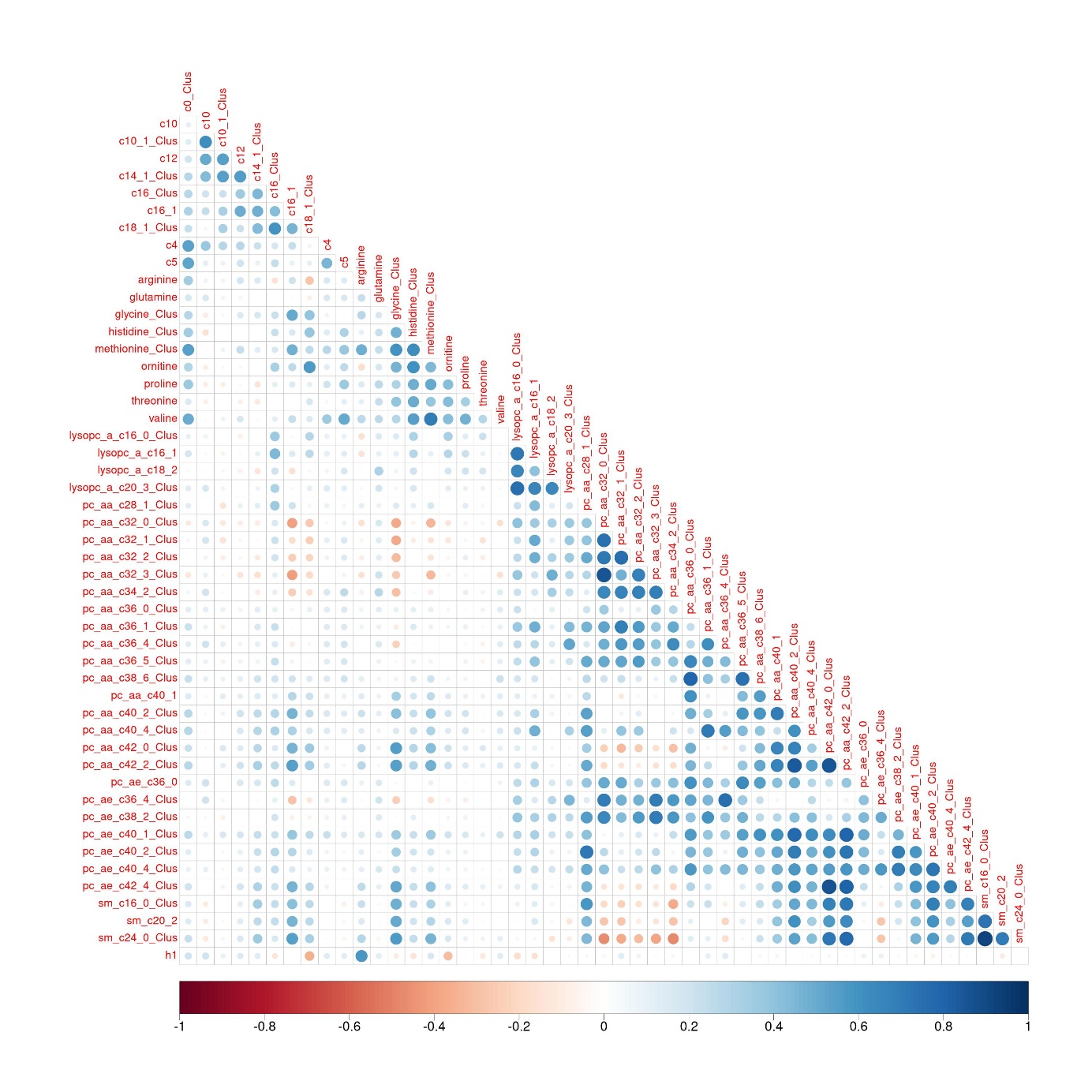


**Figure S8.** Pearson correlation between the 50 features computed in the 5,828 controls of the eight cancer type-specific EPIC studies. The 50 features correspond to 17 isolated metabolites and 33 representatives of clusters of strongly-correlated metabolites, obtained by hierarchical clustering of the 117 metabolites among the 5,828 controls. Clusters are labelled as “metabo_Clus”, with “metabo” being one of the metabolites that compose that cluster.

**
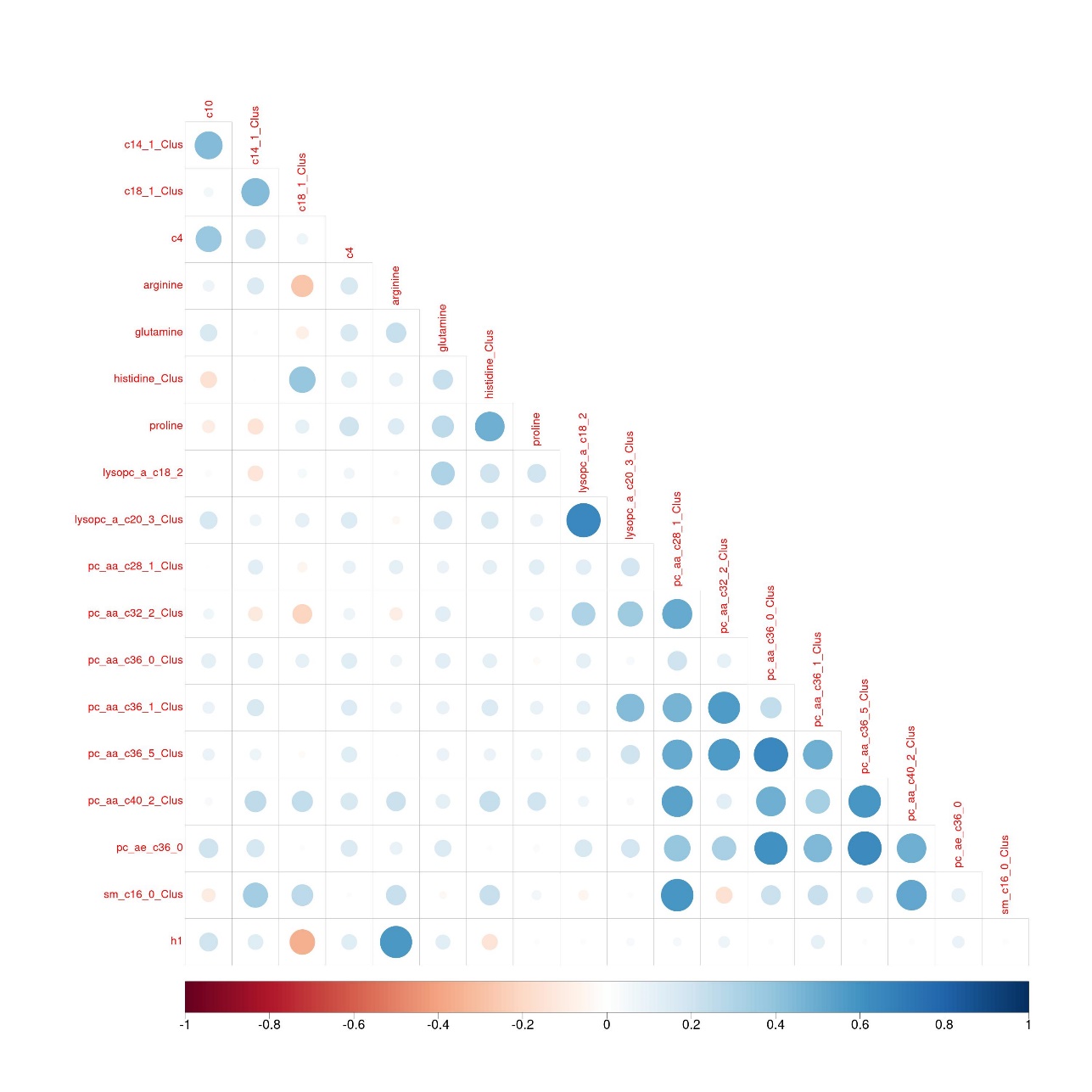
**

**Figure S9.** Pearson correlation between the 19 features related to at least one cancer site in our main analysis. Clusters are labelled as “metabo_Clus”, with “metabo” being one of the metabolites that compose that cluster.

**Table S1.** List of the 117 metabolites studied in the main analysis, and the 16 additional metabolites studied in the sensitivity analysis, following the exclusion of the participants from the second CRC study.

| **Class** | **SubClass** | **Compounds** | **Name** |
| --- | --- | --- | --- |
| **Metabolites used in the main analysis** | | | |
| acylcarnitines | acylcarnitines | C0 | Carnitine |
| acylcarnitines | acylcarnitines | C10 | Decanoylcarnitine |
| acylcarnitines | acylcarnitines | C10_1 | Decenoylcarnitine |
| acylcarnitines | acylcarnitines | C12 | Dodecanoylcarnitine |
| acylcarnitines | acylcarnitines | C14_1 | Tetradecenoylcarnitine |
| acylcarnitines | acylcarnitines | C14_2 | Tetradecadienylcarnitine |
| acylcarnitines | acylcarnitines | C16 | Hexadecanoylcarnitine |
| acylcarnitines | acylcarnitines | C16_1 | Hexadecenoylcarnitine |
| acylcarnitines | acylcarnitines | C18 | Octadecanoylcarnitine |
| acylcarnitines | acylcarnitines | C18_1 | Octadecenoylcarnitine |
| acylcarnitines | acylcarnitines | C18_2 | Octadecadienylcarnitine |
| acylcarnitines | acylcarnitines | C2 | Acetylcarnitine |
| acylcarnitines | acylcarnitines | C3 | Propionylcarnitine |
| acylcarnitines | acylcarnitines | C4 | Butyrylcarnitine |
| acylcarnitines | acylcarnitines | C5 | Valerylcarnitine |
| aminoacids | non-essential aa | Arg | Arginine |
| aminoacids | non-essential aa | Gln | Glutamine |
| aminoacids | non-essential aa | Gly | Glycine |
| aminoacids | essential aa | His | Histidine |
| aminoacids | essential aa | Met | Methionine |
| aminoacids | non-essential aa | Orn | Ornithine |
| aminoacids | essential aa | Phe | Phenylalanine |
| aminoacids | non-essential aa | Pro | Proline |
| aminoacids | non-essential aa | Ser | Serine |
| aminoacids | essential aa | Thr | Threonine |
| aminoacids | essential aa | Trp | Tryptophan |
| aminoacids | non-essential aa | Tyr | Tyrosine |
| aminoacids | essential aa | Val | Valine |
| glycerophospholipids | saturated | lysoPC_a_C16_0 | lysoPC a C16:0 |
| glycerophospholipids | mono-unsaturated | lysoPC_a_C16_1 | lysoPC a C16:1 |
| glycerophospholipids | saturated | lysoPC_a_C17_0 | lysoPC a C17:0 |
| glycerophospholipids | saturated | lysoPC_a_C18_0 | lysoPC a C18:0 |
| glycerophospholipids | mono-unsaturated | lysoPC_a_C18_1 | lysoPC a C18:1 |
| glycerophospholipids | poly-unsaturated | lysoPC_a_C18_2 | lysoPC a C18:2 |
| glycerophospholipids | poly-unsaturated | lysoPC_a_C20_3 | lysoPC a C20:3 |
| glycerophospholipids | poly-unsaturated | lysoPC_a_C20_4 | lysoPC a C20:4 |
| glycerophospholipids | mono-unsaturated | PC_aa_C28_1 | PC aa C28:1 |
| glycerophospholipids | saturated | PC_aa_C30_0 | PC aa C30:0 |
| glycerophospholipids | saturated | PC_aa_C32_0 | PC aa C32:0 |
| glycerophospholipids | mono-unsaturated | PC_aa_C32_1 | PC aa C32:1 |
| glycerophospholipids | poly-unsaturated | PC_aa_C32_2 | PC aa C32:2 |
| glycerophospholipids | poly-unsaturated | PC_aa_C32_3 | PC aa C32:3 |
| glycerophospholipids | mono-unsaturated | PC_aa_C34_1 | PC aa C34:1 |
| glycerophospholipids | poly-unsaturated | PC_aa_C34_2 | PC aa C34:2 |
| glycerophospholipids | poly-unsaturated | PC_aa_C34_3 | PC aa C34:3 |
| glycerophospholipids | poly-unsaturated | PC_aa_C34_4 | PC aa C34:4 |
| glycerophospholipids | saturated | PC_aa_C36_0 | PC aa C36:0 |
| glycerophospholipids | mono-unsaturated | PC_aa_C36_1 | PC aa C36:1 |
| glycerophospholipids | poly-unsaturated | PC_aa_C36_2 | PC aa C36:2 |
| glycerophospholipids | poly-unsaturated | PC_aa_C36_3 | PC aa C36:3 |
| glycerophospholipids | poly-unsaturated | PC_aa_C36_4 | PC aa C36:4 |
| glycerophospholipids | poly-unsaturated | PC_aa_C36_5 | PC aa C36:5 |
| glycerophospholipids | poly-unsaturated | PC_aa_C36_6 | PC aa C36:6 |
| glycerophospholipids | saturated | PC_aa_C38_0 | PC aa C38:0 |
| glycerophospholipids | poly-unsaturated | PC_aa_C38_3 | PC aa C38:3 |
| glycerophospholipids | poly-unsaturated | PC_aa_C38_4 | PC aa C38:4 |
| glycerophospholipids | poly-unsaturated | PC_aa_C38_5 | PC aa C38:5 |
| glycerophospholipids | poly-unsaturated | PC_aa_C38_6 | PC aa C38:6 |
| glycerophospholipids | mono-unsaturated | PC_aa_C40_1 | PC aa C40:1 |
| glycerophospholipids | poly-unsaturated | PC_aa_C40_2 | PC aa C40:2 |
| glycerophospholipids | poly-unsaturated | PC_aa_C40_3 | PC aa C40:3 |
| glycerophospholipids | poly-unsaturated | PC_aa_C40_4 | PC aa C40:4 |
| glycerophospholipids | poly-unsaturated | PC_aa_C40_5 | PC aa C40:5 |
| glycerophospholipids | poly-unsaturated | PC_aa_C40_6 | PC aa C40:6 |
| glycerophospholipids | saturated | PC_aa_C42_0 | PC aa C42:0 |
| glycerophospholipids | mono-unsaturated | PC_aa_C42_1 | PC aa C42:1 |
| glycerophospholipids | poly-unsaturated | PC_aa_C42_2 | PC aa C42:2 |
| glycerophospholipids | poly-unsaturated | PC_aa_C42_4 | PC aa C42:4 |
| glycerophospholipids | poly-unsaturated | PC_aa_C42_5 | PC aa C42:5 |
| glycerophospholipids | poly-unsaturated | PC_aa_C42_6 | PC aa C42:6 |
| glycerophospholipids | saturated | PC_ae_C30_0 | PC ae C30:0 |
| glycerophospholipids | mono-unsaturated | PC_ae_C32_1 | PC ae C32:1 |
| glycerophospholipids | poly-unsaturated | PC_ae_C32_2 | PC ae C32:2 |
| glycerophospholipids | saturated | PC_ae_C34_0 | PC ae C34:0 |
| glycerophospholipids | mono-unsaturated | PC_ae_C34_1 | PC ae C34:1 |
| glycerophospholipids | poly-unsaturated | PC_ae_C34_2 | PC ae C34:2 |
| glycerophospholipids | poly-unsaturated | PC_ae_C34_3 | PC ae C34:3 |
| glycerophospholipids | saturated | PC_ae_C36_0 | PC ae C36:0 |
| glycerophospholipids | mono-unsaturated | PC_ae_C36_1 | PC ae C36:1 |
| glycerophospholipids | poly-unsaturated | PC_ae_C36_2 | PC ae C36:2 |
| glycerophospholipids | poly-unsaturated | PC_ae_C36_3 | PC ae C36:3 |
| glycerophospholipids | poly-unsaturated | PC_ae_C36_4 | PC ae C36:4 |
| glycerophospholipids | poly-unsaturated | PC_ae_C36_5 | PC ae C36:5 |
| glycerophospholipids | saturated | PC_ae_C38_0 | PC ae C38:0 |
| glycerophospholipids | poly-unsaturated | PC_ae_C38_2 | PC ae C38:2 |
| glycerophospholipids | poly-unsaturated | PC_ae_C38_3 | PC ae C38:3 |
| glycerophospholipids | poly-unsaturated | PC_ae_C38_4 | PC ae C38:4 |
| glycerophospholipids | poly-unsaturated | PC_ae_C38_5 | PC ae C38:5 |
| glycerophospholipids | poly-unsaturated | PC_ae_C38_6 | PC ae C38:6 |
| glycerophospholipids | mono-unsaturated | PC_ae_C40_1 | PC ae C40:1 |
| glycerophospholipids | poly-unsaturated | PC_ae_C40_2 | PC ae C40:2 |
| glycerophospholipids | poly-unsaturated | PC_ae_C40_3 | PC ae C40:3 |
| glycerophospholipids | poly-unsaturated | PC_ae_C40_4 | PC ae C40:4 |
| glycerophospholipids | poly-unsaturated | PC_ae_C40_5 | PC ae C40:5 |
| glycerophospholipids | poly-unsaturated | PC_ae_C40_6 | PC ae C40:6 |
| glycerophospholipids | mono-unsaturated | PC_ae_C42_1 | PC ae C42:1 |
| glycerophospholipids | poly-unsaturated | PC_ae_C42_2 | PC ae C42:2 |
| glycerophospholipids | poly-unsaturated | PC_ae_C42_3 | PC ae C42:3 |
| glycerophospholipids | poly-unsaturated | PC_ae_C42_4 | PC ae C42:4 |
| glycerophospholipids | poly-unsaturated | PC_ae_C42_5 | PC ae C42:5 |
| glycerophospholipids | poly-unsaturated | PC_ae_C44_3 | PC ae C44:3 |
| glycerophospholipids | poly-unsaturated | PC_ae_C44_4 | PC ae C44:4 |
| glycerophospholipids | poly-unsaturated | PC_ae_C44_5 | PC ae C44:5 |
| glycerophospholipids | poly-unsaturated | PC_ae_C44_6 | PC ae C44:6 |
| sphingolipids | sphingomyelin | SM_OH_C14_1 | SM (OH) C14:1 |
| sphingolipids | sphingomyelin | SM_OH_C16_1 | SM (OH) C16:1 |
| sphingolipids | sphingomyelin | SM_OH_C22_1 | SM (OH) C22:1 |
| sphingolipids | sphingomyelin | SM_OH_C22_2 | SM (OH) C22:2 |
| sphingolipids | sphingomyelin | SM_OH_C24_1 | SM (OH) C24:1 |
| sphingolipids | sphingomyelin | SM_C16_0 | SM C16:0 |
| sphingolipids | sphingomyelin | SM_C16_1 | SM C16:1 |
| sphingolipids | sphingomyelin | SM_C18_0 | SM C18:0 |
| sphingolipids | sphingomyelin | SM_C18_1 | SM C18:1 |
| sphingolipids | sphingomyelin | SM_C20_2 | SM C20:2 |
| sphingolipids | sphingomyelin | SM_C24_0 | SM C24:0 |
| sphingolipids | sphingomyelin | SM_C24_1 | SM C24:1 |
| sugars | sugars | H1 | Sum of Hexoses |
| **Additional metabolites used in the analysis following the exclusion of the second CRC sub-study** | | | |
| aminoacids | non-essential aa | Ala | Alanine |
| aminoacids | non-essential aa | Asn | Asparagine |
| aminoacids | non-essential aa | Asp | Aspartate |
| aminoacids | non-essential aa | Cit | Citrulline |
| aminoacids | non-essential aa | Glu | Glutamate |
| aminoacids | essential aa | Ile | Isoleucine |
| aminoacids | essential aa | Leu | Leucine |
| aminoacids | essential aa | Lys | Lysine |
| biogenic amines | biogenic amines | alpha_AAA | alpha-Aminoadipic acid |
| biogenic amines | biogenic amines | Creatinine | Creatinine |
| biogenic amines | biogenic amines | Kynurenine | Kynurenine |
| biogenic amines | biogenic amines | Serotonin | Serotonin |
| biogenic amines | biogenic amines | Spermidine | Spermidine |
| biogenic amines | biogenic amines | Spermine | Spermine |
| biogenic amines | biogenic amines | Taurine | Taurine |
| glycerophospholipids | poly-unsaturated | PC_ae_C30_2 | PC ae C30:2 |

**Table S2.** Robustness of the associations identified in the main analysis when including all the pairs from the prostate cancer study. Bootstrap samples were generated from the sample comprising 6,992 matched case-control pairs after including the 1,164 pairs from the prostate cancer study with missing information on tumour stage. Those pairs were labelled as ‘Unknown PrC’. For each identified association, the proportion of bootstrap samples on which it was replicated is reported (in bold when $\geq$50%). BrC stands for breast cancer, CRC for colorectal cancer, EnC for endometrial cancer, HCC for hepatocellular carcinoma, and Adv.PrC Loc.PrC for advanced and localized prostate cancers, respectively.

| **Feature** | **Cancer Type^*^** | **Proportion of bootstrap samples** |
| --- | --- | --- |
| **Overall associations with cancer risk** | | |
| c10 | Overall | 44% |
| c4 | Overall | 41% |
| glutamine | Overall | **86%** |
| proline | Overall | **58%** |
| lysopc_a_c18_2 | Overall | **54%** |
| pc_aa_c28_1_Clus | Overall | 32% |
| pc_aa_c32_2_Clus | Overall | 47% |
| pc_aa_c36_0_Clus | Overall | **94%** |
| pc_aa_c36_1_Clus | Overall | **80%** |
| **Cancer type-specific associations** | | |
| c14_1_Clus | BrC | **79%** |
| proline | BrC | **65%** |
| pc_aa_c36_5_Clus | BrC | 41% |
| arginine | CRC | **72%** |
| histidine_Clus | CRC | **79%** |
| pc_ae_c36_0 | CRC | **75%** |
| sm_c16_0_Clus | EnC | **88%** |
| lysopc_a_c20_3_Clus | HCC | 25% |
| pc_aa_c40_2_Clus | HCC | **59%** |
| sm_c16_0_Clus | HCC | **90%** |
| c18_1_Clus | Adv.PrC | **58%** |
| lysopc_a_c18_2 | Loc.PrC | 12% |
| pc_aa_c36_0_Clus | Loc.PrC | 45% |
| h1 | Loc.PrC | **79%** |

**Table S3.** Other associations identified in a large proportion of bootstrap samples when including all the pairs from the prostate cancer study. Bootstrap samples were generated from the sample comprising 6,992 matched case-control pairs after including the 1,164 pairs from the prostate cancer study with missing information on tumour stage. Those pairs were labelled as ‘Unknown PrC’. Associations identified in at least 50% of both bootstrap analyses are reported, along with the proportion of bootstrap samples in which they were identified, and the corresponding average log odds-ratio (as estimated by the data shared lasso on each bootstrap sample). BrC stands for breast cancer, CRC for colorectal cancer, EnC for endometrial cancer, HCC for hepatocellular carcinoma, and Adv.PrC , Unk.PrC, Loc.PrC for advanced, unknown and localized prostate cancers, respectively.

| **Feature** | **Cancer Type^*^** | **Proportion of bootstrap samples^2^** | **Average log-OR^2^** |
| --- | --- | --- | --- |
| **Overall associations with cancer risk** | | | |
| valine | Overall | 56% | 0.04 |
| lysopc_a_c16_0_Clus | Overall | 58% | -0.07 |
| pc_aa_c40_1 | Overall | 65% | 0.04 |
| pc_ae_c36_0 | Overall | 69% | 0.07 |
| **Type-specific associations** | | | |
| c10 | BrC | 58% | 0.11 |
| c5 | BrC | 59% | -0.08 |
| arginine | BrC | 67% | -0.15 |
| pc_aa_c32_3_Clus | CRC | 57% | -0.19 |
| pc_aa_c34_2_Clus | CRC | 61% | 0.15 |
| pc_aa_c40_2_Clus | CRC | 60% | -0.14 |
| glycine_Clus | EnC | 76% | -0.18 |
| c10_1_Clus | EnC | 52% | -0.18 |
| arginine | KiC | 69% | 0.21 |
| lysopc_a_c18_2 | KiC | 55% | -0.20 |
| pc_aa_c36_5_Clus | GBC | 71% | -0.82 |
| pc_aa_c36_5_Clus | HCC | 81% | -0.65 |
| pc_aa_c42_2_Clus | Adv.PrC | 62% | -0.38 |
| c10 | Unk.PrC | 80% | -0.15 |
| c16_1 | Loc.PrC | 55% | -0.15 |
| lysopc_a_c16_0_Clus | Loc.PrC | 88% | 0.25 |
| lysopc_a_c16_1 | Loc.PrC | 91% | -0.22 |
| pc_ae_c40_2_Clus | Loc.PrC | 54% | -0.13 |
